# A quantitative comparative analysis of the *Four Medical Tantras* of Tibetan medicine and the *As⋅ṭā $\dot{n}$ ga Hṛdaya Saṃhitā* of Ayurveda: a focus on diet, medicinal materials, and preparations

**DOI:** 10.3389/fphar.2025.1595646

**Published:** 2025-09-02

**Authors:** Zhuoma Garang, Gebai Zhaxi, Zhuoma Suonan, Mejia La, Rizhun Luo, Bai Bai, Lame Lizhen, Jingwen Zhang, Tsedien Nhamdriel, Silang Jiangyong, Yongzhong Zeweng, Ma Mi, Zhang Wang

**Affiliations:** ^1^ College of Ethnomedicine, Chengdu University of Traditional Chinese Medicine, Chengdu, China; ^2^ School of Foreign Language and Literature, Southwest Minzu University, Chengdu, China; ^3^ The Department of Tibetan Medicine, Xizang University of Tibetan Medicine, Lhasa, China; ^4^ National Inheritance Studio for Famous Doctor of Traditional Chinese Medicine (Tibetan Doctor, Jiangyong Silang), Chengdu University of Traditional Chinese Medicine, Chengdu, China; ^5^ Research Institute of Traditional Indian Medicine, Chengdu University of Traditional Chinese Medicine, Chengdu, China

**Keywords:** Tibetan medicine, *Four Medical Tantras*, Ayurveda, *As⋅ṭān⋅ga Hṛdaya Saṃhitā*, foods, drinks, medicinal materials, preparations

## Abstract

**Ethnopharmacological relevance:**

In 2023, the classic work of Tibetan medicine, the *Four Medical Tantras*, was successfully inscribed on the Memory of the World Register. The *As⋅ṭā $\dot{n}$ ga Hṛdaya Saṃhitā* was also introduced to Tibet in the 10th century A.D. and translated into Tibetan. Although many scholars at home and abroad have shown interest in the historical exchange between the two texts, no quantitative and objective comparative research results have yet been published.

**Aim of the study:**

This study aims to reveal and compare the use of diets, medicinal materials and preparations as therapeutic means in the *Four Medical Tantras* and the *As⋅ṭā $\dot{n}$ ga Hṛdaya Saṃhitā*, and to explore their historical exchanges between these two traditional medical system.

**Materials and methods:**

① Data mining: Relevant information on diets, medicinal materials, and preparations was extracted from the *Four Medical Tantras* and the *As⋅ṭā $\dot{n}$ ga Hṛdaya Saṃhitā*, and entered into a Microsoft Excel 2010 table to establish the datasets, respectively. ② Statistical analysis: IBM SPSS statistics 27.0, SPSS Modeler 18.0 and Gephi 0.9.2 were used to analyze the contents of diets, medicinal materials, and preparations, such as frequency, association rules, and complex networks. ③ Comparative study: The Mann-Whitney test, a non-parametric method, and the intuitive comparison method were used to analyze the similarities and differences in terms of therapeutic means of diets, medicinal materials, and preparations documented in the *Four Medical Tantras* and the *As⋅ṭā $\dot{n}$ ga Hṛdaya Saṃhitā*.

**Results:**

① Foods: The *Four Medical Tantras* document 153 kinds, categorized into five major types, mainly meat. The *As⋅ṭā $\dot{n}$ ga Hṛdaya Saṃhitā* documents 392 types, categorized into six major types, mainly vegetables. There are 49 shared food types between the two texts. ② Drinks: The *Four Medical Tantras* document 65 kinds, categorized into three major types. The *As⋅ṭā $\dot{n}$ ga Hṛdaya Saṃhitā* documents 81 kinds, categorized into five major types (including more sugar cane- and sesame oil-based types), and includes South Asian specialty drinks such as yellow cow urine, camel urine, and elephant urine. Among them, 18 drink types are shared. ③ Medicinal materials: The *Four Medical Tantras* document 1,115 species, mainly animal-based medicines, with 388 recorded efficacies classified into 17 disease types. The *As⋅ṭā $\dot{n}$ ga Hṛdaya Saṃhitā* documents 762 species, mainly plant-based, with 40 recorded efficacies classified into 30 disease types. Among them, 227 medicinal materials are shared, with 21 overlapping recorded efficacies. ④ Preparations: The *Four Medical Tantras* document 2,678 types, with combinations following the principle of “coolness in warmth, and warmth in coolness,” reflecting Tibetan medical characteristics. The *As⋅ṭā $\dot{n}$ ga Hṛdaya Saṃhitā* documents 2,240 types, commonly using pepper, ghee, and honey, embodying Ayurveda’s principle of “combining food and medicine” and reflecting its characteristics approach to medication.

**Conclusion:**

This study found both differences and commonalities in their therapeutic content. The both texts differ markedly in the total amount and classification of therapeutic content, with particularly striking differences in dietary types and sources of medicinal materials, reflecting the different ecological and cultural characteristics of the Tibetan Plateau and South Asia. At the same time, there is a partial overlap between the both texts, such as 9.9% of foods, 14.1% of drinks, and 13.8% of medicinal materials are shared, with a 48% similarity in their recorded efficacies. These findings provide an empirical basis for understanding the similarities and differences between Tibetan medicine and the Ayurvedic medical system, and open new perspectives for comparative studies of traditional medicine.

## Graphical Abstract

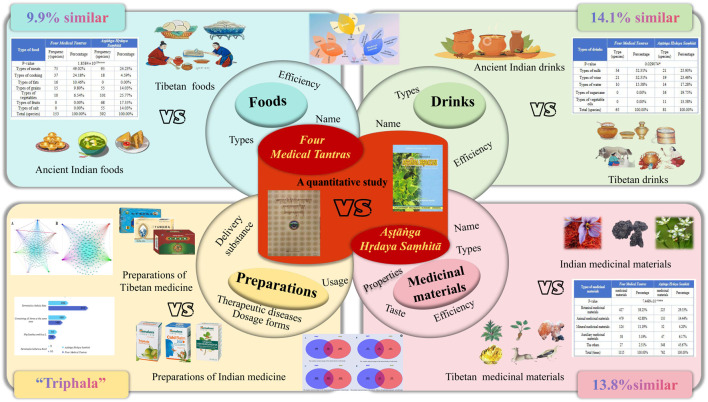

## 1 Introduction

The *Four Medical Tantras* (Tibetan name: དཔལ་ལྡན་རྒྱུད་བཞི།) is the cornerstone of Tibetan medicine and was compiled by Yutuo Nyingma Yundan Gonpo at the end of the eighth century A.D., and was finalized and handed down to the present day by the revisions and additions made by Yutuo Sama Yundan Gonpo in the 12th century A.D. (Dorje, 2022). The original text is written in Tibetan with prose and rhyme. It consists of four parts: Root Treatise, Explanatory Treatise, Oral Instruction Treatise, and Final Treatise (Jingfeng, 2002), with a total of 156 chapters, categorized as the “Eight Branches,” i.e., the “Body Branch, Children’s Branch, Women’s Branch, Evil Branch, Trauma Branch, Poisoning Branch, Pension Branch, and Nourishing Branch.” The book is a collection of practical and theoretical essence from one of the classic treatises on Tibetan medicine, known as the Tibetan medicine “encyclopedia” (Newsletter, 2023). It has ancient and modern annotations totaling more than one hundred (Cairang et al., 2022). Among them, *Ancestors’ Oral Recordings*, *Blue Liuli*, and *Great Detailed Explanation of the Four Medical Tantras* are the authoritative works on the full annotated text of the *Four Medical Tantras* (Dengdu, 2015b). It has been translated into Mongolian, Chinese, English, German, Russian, Japanese, and other languages (Cuomu, 2023; Jiayin, 2021). The book is one of the most complete and influential representatives of traditional medicine and is an essential document of great cultural, historical, and research value (Dangzhi et al., 2019). On 24 May 2023, the *Four Medical Tantras* was successfully selected for the Memory of the World (International) Register (Cuomu, 2023), marking the fact that Tibetan medicine and the culture it carries are moving towards a broader international perspective. It is significant to spread and practice the spirit of Tibetan medicine to help the world.

The *As⋅ṭāṅga Hṛdaya Saṃhitā* (Sanskrit name: अष्टाङ्गहृदयसंहिता) is the most classical and central work of Ayurveda, which, along with the ancient Indian medical works, the *Susruta Saṃhitā* and the *Charaka Saṃhitā,* is known as the *Brihat Trayee* of ancient Vedic medicine (Jain, 1980). It was written around the 6th– centuries AD by Vagbhata (Mukherjee et al., 2017). The book mainly synthesizes and draws on the essence of the two earliest medical classics of India, the *Susruta Saṃhitā* (which primarily deals with surgery) and the *Charaka Saṃhitā* (which mainly deals with oral administration medicine) (Vidyanath and Niṣṭēśvar, 2015). The original text of this book is written in the ancient Sanskrit rhyming style, and the whole text consists of six parts, namely, Sutrasthana, Sarirasthana, Nidanasthana, Cikitsasthana, Kalpasiddhisthana, and Uttarasthana. It consists of 120 chapters, which are categorized into “eight branches,” namely, Kaya (Oral administration or general medicine), Dala (pediatrics), Graha (demonology, possession by evil spirits or psychosomatic), Urdhvanga (diseases of parts head), Salya (surgery or removal of foreign bodies), Damstra (toxicology), Jara (geriatrics) and Vrsa (branch dealing with virility). At present, there are as many as 30 kinds of annotations, and seven kinds of total or residual copies exist (Krs, 2016), among which the *Self-Annotations of As⋅ṭāṅga Hṛdaya Saṃhitā* and the *As⋅ṭāṅga Hṛdaya Saṃhitā Annotations - Moonlight* on Words and Meanings were included in the 13th century in the *Bstan’ your* section of the *Ta-ts’ang Ching*, which is preserved intact up to the present day. The book has been translated into Arabic, Tibetan, German, English, and Mongolian (Krs, 2016). In China, there are already Tibetan and Mongolian versions of the book. According to the literature, the Tibetan version was translated from Sanskrit into Tibetan in the early 10th century CE by the Indian Jarandhara and the Tibetan master translator Rinchen Sangpo. It was included in the 13th century in the *Bstan’ gyur* section of the *Ta-ts’ang Ching* (Tsongkha, 2006). The Mongolian version was printed in Beijing in the 18th century based on the Tibetan translation (Nashun and Geri, 2005).

Tibetan medicine and Ayurveda both play an essential role in traditional medicine, and both have a long history and a unique system of medicine. The development of Tibetan medicine has gone through six crucial stages: germination, foundation, growth, controversy, prosperity, and revitalization (Jingfeng, 2002). It has interacted and exchanged with other medicines during its development and evolution. In the process of the development of Tibetan medicine, there have been certain exchanges between “Seljuks” (Arabian) medicine, “Sindhu” (Indian) medicine, and “Datang” (Chinese) medicine, of which Indian medicine had the earliest exchanges with Tibetan medicine due to geography and the spread of Buddhist culture and other factors. The Kailas Range in Tibet is also held as a sacred mountain for Hinduism, and especially after Buddhism in India spread to Tibet, the interaction between Tibet and India became much closer, and even many well-known Tibetan medical doctors visited India as a way to increase their reputation (Dengdu, 2015a). In the seventh century A.D., with the spread of Buddhism, many Indian medical texts were translated, contributing to the maturation of Tibetan medical science. At that time, the Indian medical masterpieces that were introduced into Tibet included the *As⋅ṭāṅga Hṛdaya Saṃhitā* (ཡན་ལག་བརྒྱད་པའི་སྙིང་པོ་བསྡུས་པ།), *Hundred Theories of Medical Formulas* (སྦྱོར་བ་བརྒྱ་པ།), *Medical Scriptures* (འཚོ་བའི་མདོ།), *As⋅ṭāṅga Hṛdaya Saṃhitā- Explaining Moonlight* (ཡན་ལག་བརྒྱད་པའི་སྙིང་པོ་བསྡུས་པའི་རྒྱས་འགྲེལ་ཟླ་བའི་འོད་ཟེར།), and *Eight Medicinal Names of Medicine* (སྨན་མིང་ཟུར་བཀོལ་སྒྲ་སྦྱོར་ངེས་པ།), which were subsequently incorporated into the Buddhist classic *Bstan’ gyur*, and which had a certain impact on the formation and development of the theoretical system of Tibetan medicine (Dengdu, 2015b).

Many scholars at home and abroad have conducted extensive and in-depth research on the relationship between Tibetan medicine and Ayurveda from different academic perspectives at different times, among which the research of Dr. Mariana Wendell and others has a certain degree of representatives and influence. Her study of Tibetan medicine and Ayurveda in terms of fluids pathology, pathogenesis, disease diagnosis and physiological understanding showed that there were many commonalities between the two and indicated that the connection was not purely accidental and that evidence of a direct or indirect link between the two classical medicines should be found and deserve continued attention (Winde and Tiechen, 2011; Joseph, 1998; Joseph, 2009; Allen, 2001). Scholars such as Sun (2019) and Li (2025) have comparatively analyzed the pharmaceutical preparations of Indian Ayurveda and Chinese traditional medicine (including Tibetan medicine), while Li Xiaoli (Lixiao, 2021) and Zhang Jingwen (Zhangjing, 2023) have focused on the differences in the medicinal characteristics and therapeutic applications of medicinal materials between China and India. The results of this study show that although there are some similarities between the two systems of medicine in terms of the efficacy of medicines, the selection of medicinal materials, and the way of combining them, reflecting the possibility of direct or indirect historical exchanges between Chinese and Indian traditional medicine, there are significant differences between the two systems of medicine in terms of medicinal material preparation, mainly in the structure of the main diseases, the variety of medicinal materials, and the pattern of combining them. This difference may be due to the different influences of local climate, geographic environment, and material resources.

In the traditional medical system, the *Four Medical Tantras* and the *As⋅ṭāṅga Hṛdaya Saṃhitā* have an important academic status as representative classical works of Tibetan and Ayurveda medicine, respectively (Ciren, 2020). It is worth noting that some scholars have suggested that the *Four Medical Tantras* may have borrowed some of the contents of the *As⋅ṭāṅga Hṛdaya Saṃhitā*, a view that has triggered widespread concern in the academic community. Based on this, scholars at home and abroad have launched a multi-dimensional comparative study of the intrinsic relevance of the two classic works and their specific contents from different research perspectives. German scholar R.E. Emmerick compared the contents of epilepsy and geriatrics in the *Four Medical Tantras* with the *As⋅ṭāṅga Hṛdaya Saṃhitā*, and finally concluded that there were both similarities and differences between the two medical books (Ga, 2015). Chinese scholars, such as Cai Jingfeng and Hong Wuwu, briefly compare and contrast the contents of the chapters and “eight branches”, diagnosis and treatment of the *Four Medical Tantras* and the *As⋅ṭāṅga Hṛdaya Saṃhitā*, and propose the similarities and differences (Jingfeng and Wulei, 1999). Liu Yinghua and Li Na also discussed the “eight branches” method of disease division used in these two medical books, and after comparing them, found that gynecology and the five senses were recorded separately in the *Four Medical Tantras*, while the division method was not found in the *As⋅ṭāṅga Hṛdaya Saṃhitā*, and concluded that there was a difference in the “eight branches” division method between the two (Yinghua and Na, 2009). Li Xianjia et al. compared the contents of the framework of the entire book of the *As⋅ṭāṅga Hṛdaya Saṃhitā* and the *Four Medical Tantras* and presented the similarities and differences between these two medical works (Xianjia and Duojie, 2020). Rinzin et al. compare and contrast some of the theories and core concepts of the *Susruta Saṃhitā*, the *As⋅ṭāṅga Hṛdaya Saṃhitā*, and the *Four Medical Tantras* to examine the connections between these three works (Renzeng, 2018). Luo Rizhun and other research shows that although both texts use the diagnostic methods of looking, asking and touching, the diagnostic system of the *Four Medical Tantras* is more complete, especially characterized by urological diagnosis; in contrast, the *As⋅ṭāṅga Hṛdaya Saṃhitā* focuses more on visual diagnosis and does not deal with the content of pulse diagnosis (Luori, 2023). A comparative study of basic theories and external therapies by Zazhi Gebai and others found that both texts share commonalities in the core theoretical frameworks of the three elements and the five sources, but differ significantly in their specific theoretical constructs: the *As⋅ṭāṅga Hṛdaya Saṃhitā* emphasizes the connection between the three elements and the five realms and the five colors, while the *Four Medical Tantras* develops a unique theoretical system of “Ignorance-Three Poisons-Three Causes”, and its doctrine of the five sources is also more detailed (Zhaxi, 2025). To summarize, most of the existing studies have focused on the qualitative analysis of the chapter structure, basic theories and diagnostic and therapeutic methods of the both texts, which have initially revealed the similarities and differences between the two systems, but the systematic comparative study of the dietary therapies, the application of medicinal materials, and the preparation process and other specific therapeutic contents are still lacking. This research gap needs to be filled in order to understand more comprehensively the intrinsic connection and characteristic differences between the two systems of medicine.

As the classic representatives of Tibetan medicine and Ayurveda medicine, both the *Four Medical Tantras* and the *As⋅ṭāṅga Hṛdaya Saṃhitā* list dietary and pharmacological therapies as important therapeutic means, and the relevant contents are found throughout the main chapters of both texts, which are of significant research value and academic representatives. This study takes the food, drinks, medicinal materials, and preparations included in the *Four Medical Tantras* and the *As⋅ṭāṅga Hṛdaya Saṃhitā* as the research objects, and adopts the methods of frequency analysis, association rule analysis and complex network analysis, combined with non-parametric test (Mann-Whitney U test), to systematically compare the differences and similarities between the both texts from the therapeutic point of view. By exploring the differences and complementarities in the therapeutic contents of the both texts and sorting out the characteristics and laws of their therapeutic contents, we can then reveal the cultural and philosophical connotations of Tibetan medicine and Ayurveda medicine in dietary therapies and medicinal materials therapies, and explore their cross-cultural exchanges and historical origins.

## 2 Methodology

### 2.1 Literature sources

In the *Four Medical Tantras* (Gunbo, 2018) (Tibetan Dege Edition), diet, medicinal materials, and preparations make up a considerable proportion of the content. For example, the *Root Treatise* contains 1/6 of the chapters. The *Explanatory Treatise* contains 14/31 of the chapter content. The *Oral Instruction Treatise* contains 90/92 of the chapters. The *Final Treatise* contains 23/25 chapters, so that in the entire 156 chapters of the book, the proportion of therapeutic chapters amounts to 82%.

The *As⋅ṭāṅga Hṛdaya Saṃhitā* (Bata, 2013) (Tibetan Dege Edition, translated by Master Rinchen Sangpo) covers a great deal of ground on diets, medicinal materials, and preparations in its pages. For example, the Sutrasthana contains 24/30 chapters. The Sarirasthana consists of 2/6 chapters. The Cikitsasthana consists of 22/22 chapters. And the Kalpasiddhisthana is divided into 6/6 chapters. The Uttarasthana contains 27/40 chapters, making a total of 120 chapters in the book, of which the healing section accounts for 68%.

### 2.2 Information extraction and creation of data sets

Information was extracted related to diets, medicinal materials, and preparations from the *Four Medical Tantras* and the *As⋅ṭāṅga Hṛdaya Saṃhitā*, including various names and types of diets. This included information about medicinal materials, such as their names, quantities, types, properties, and methods of categorization. It also included multiple aspects of preparations (such as quantities, dosage forms, taste quantities, modes of use, substances for delivery, and diseases treated), all of which were entered into the Microsoft Excel 2010 to create appropriate information datasets for foods, drinks, medicinal materials, and preparations.

### 2.3 Data normalization process

In this study, the main reference in the data mining and cleaning process are the *Chinese Pharmacopoeia* (National Pharmacopoeia Commission, 2020), *Four Continuations of Medicine* (Gunbo and Yixue, 2012), *Four Medical Classics* (Gunbo, 2019), *Tibetan Medicine Mirror Crystal medicinal material* (Gawu, 2018) (Tibetan), *Tibetan-Chinese Contrasting Nomenclature of Tibetan Medicine* (Qinghai and Xizang, 2018), *Blue Liuli* (Jiacuo, 2005) (Tibetan), *Ancestors’ Oral Recordings* (Jiabo, 1989) (Tibetan), *Self-Explanation of As⋅ṭāṅga Hṛdaya Saṃhitā* (Literature, 2005) (Tibetan translation), *As⋅ṭāṅga Hṛdaya Saṃhitā Annotations-Moonlight* (Medicine, 2015) (Tibetan translation) and other authoritative sources. The names of diets, medicinal materials, and diseases used in this paper have been standardized. In addition, numerous specialized terms in Tibetan medicine and Ayurveda are challenging to correspond to Western medical terminology. Thus, flexible treatments such as transliteration, Italian translation, and direct translation have been adopted to avoid misunderstanding and confusion.

### 2.4 Statistical analysis

#### 2.4.1 Frequency analysis

The Excel data set of foods, drinks, medicinal materials, and preparations from the *Four Medical Tantras* and *As⋅ṭāṅga Hṛdaya Saṃhitā* was imported into IBM SPSS Statistics 27.0 for frequency analysis.

#### 2.4.2 Association rule analysis

The Excel datasets of foods, drinks, medicinal materials and preparations contained in the *Four Medical Tantras* and the *As⋅ṭāṅga Hṛdaya Saṃhitā* were processed as follows: ①Data cleansing - ②Transaction data - ③Data matrix, and the data were imported into SPSS Modeler 18 software in “.sav” or “.xlsx” format, and the “*Apriori*” algorithm was applied. Then the data were imported into SPSS Modeler 18 software in “.sav” or “.xlsx” format, and its “*Apriori*” algorithm was applied. Under the conditions of “minimum conditional support of 5%, minimum rule confidence level of 20%, and maximum number of antecedents of 3,” the study investigated the association rule for the preparation of medicinal material.

#### 2.4.3 Complex network analysis

The preparation data from the *Four Medical Tantras* and *As⋅ṭāṅga Hṛdaya Saṃhitā* were imported into Gephi 0.9.2 software as readable CSV files for cluster analysis, and the weights of the edges, which indicated the strength of association between the medicinal materials, were analyzed by the k-core method, then visualized and displayed. Gephi is an open source and free visualization software used for network analysis (Deng et al., 2014). Using the “Statistics” module of the software, the topological characteristics of the network in terms of “average degree”, “network diameter” and “eigenvector centrality” were calculated. The topological parameters “average degree,” “network diameter,” and “eigenvector centrality” are calculated (Yu et al., 2013; Yuan, et al., 2013; Ulrik, 2001; Liu and Du, 2017). Tibetan medicine and Ayurveda preparations have complex associations and unclear laws between the medicinal materials in the formulas, which are typical of complex networks. The Gephi 0.9.2 software was used to construct a complex network of the medication laws, to explore the potential laws between the medicinal materials in the formulas, and to derive an intuitive and clear complex network diagram. Node degree is the number of neighboring nodes a node has; the larger the node degree, the more important the node is. Eigenvector centrality means that it is an important node in the network and is strongly associated with its connected nodes.

#### 2.4.4 Quantitative comparative analysis

Using the Mann-Whitney test in the nonparametric tests provided by the SPSS 27.0 software, a quantitative comparative study of the *Four Medical Tantras* and the *As⋅ṭāṅga Hṛdaya Saṃhitā* regarding the types of diets, the number of diets, the number of medicinal materials, the kinds of medicinal materials, their sexual tastes, the methods of classifying medicinal materials, the number of preparations, the dosage forms of preparations, the number of medicinal taste, how preparations are used, the substances in which the preparations are delivered, and the characteristics of the preparations primarily used in treating illnesses. “*P < 0.05” indicates that the difference between means is statistically significant, “**P < 0.01” suggests that the difference between means is highly statistically significant, and “***P < 0.001” indicates that the difference between the means is highly statistically significant.

## 3 Results

### 3.1 General comparative study of diets, medicinal materials, and preparations

Through quantitative statistics on foods, drinks, medicinal materials, and preparations in the *Four Medical Tantras* and the *As⋅ṭāṅga Hṛdaya Saṃhitā*, the study’s results showed that the *Four Medical Tantras* covered 153 foods, 65 drinks, 1,115 medicinal materials, and 2,678 preparations. The *As⋅ṭāṅga Hṛdaya Saṃhitā* covered 392 foods, 81 drinks, 762 medicinal materials, and 2,240 preparations. This study shows a parameter-free test comparison and visualization comparison analysis of the therapeutic regimens such as diets, medicinal materials, and preparations derived from the above statistics.

#### 3.1.1 Overall comparison of treatments

The results of the non-parametric test showed that the therapeutic means of both texts in terms of the types of foods, types of drinks, medicinal materials that record efficacy, medicinal materials in preparations, single-prescription, and compound preparations were not similar in general (*P* < 0.05).

Through visual comparison analysis, we found that the number of treatments in both texts differed. In the *Four Medical Tantras*, 2,604 compound preparations, 74 single-prescription, 1,103 prescription medicinal materials, 338 medicinal materials that record efficacy, 153 foods, and 65 drinks are used. In the *As⋅ṭāṅga Hṛdaya Saṃhitā*, 2,093 compound preparations, 147 single-prescription, 752 prescription medicinal materials, 40 medicinal materials that record efficacy, 392 foods and 81 drinks were used. Table 1 presents the details of these two sources.

**TABLE 1:** Comparative analysis of treatment methods.

Treatment method	*Four Medical Tantras*			*As⋅ṭā $\dot{n}$ ga Hṛdaya Saṃhitā*		
	Frequency (species)	Percentage	Example	Frequency (species)	Percentage	Example
*P-value*	1.5473 × 10^−123^***					
Compound preparation	2,604	59.36%	Sanwei Guangmingyan Soup, Siwei Shaji Powder, Siwei Awei Pill, Wuwei Xinjiang Powder, Wuwei Guangmuxiang Powder, Qiwei Roudoukou Pill, Qiwei Bingpian Powder, Liuwei Shiliuzi Powder, Liuwei Kuanjintang, Bawei Zhangyacai, Erwei Tanxiang Powder, Erwei Ganqingqinglan Powder, Jiuwei Caomuli Powder, Ershiwuwei Lvxue Powder, Shiwei Tuoshou Plaster, Shiwei Hezi Plaster, Shierwei Heibingpian Pill, Shierwei Zhaxun Powder, Shiwuwei Tianzhuhuang Plaster, Shiqiwei Xiaodahuang Soup, Ershiliuwei Hezi Pill	2093	59.71%	This book has only the names of the medicinal materials that make up the formula and does not record the names of the preparations.
Single-prescription	74	1.69%	Chebulbul soup, Yuganzi soup, Zinao sha soup, dried ginger soup, duckweed soup, bright salt soup, swertia soup, celandine soup, broad-veined vine soup, coriander soup, parsley tamarind soup, and so on.	147	4.19%	This book has only the names of the medicinal materials that make up the formula and does not record the names of the preparations.
Prescription medicinal materials	1,103	25.14%	*T. chebula.*, *C. sativus*, *P. longum.*, *White sugar*, *B. concretio*, *Z. officinale.*, *S. thibetanus*, *A. craving.*, *A. cerana*, *P. emblica.*, *P. nigrum.*, *P. nigrum.*, *S. album.*, *M. fragrans*, *L. brevituba.*, *A. lappa*, *O. erythrotis*, *S. chirayita.*, *Moschusberezovskii*, *A. tanguticum*, *Sallucidu*, *Sal Ammoniac*, *Brown sugar*, *T. bellerica*, *G. uralensis.*, *B. grannies*, *H. violaceous*, *C. cassia*, *E. fischeriana*, *A. evasive*, *P. granatum.*, *Ghee*, *D. aromatica*, *B. aristata.*	752	21.46%	*P. longum*, *Ghee*, *A. cerana*, *T. chebula.*, *Z. officinale.*, *P. nigrum.,White sugar*, *T. bellerica*, *D. nobile.*, *Sesame Oil*, *G. uralensis.*, *P. emblica.*, *A. marmelos*, *C. frutescens.*, *Sallucidum*, *B. aristata.*, *S. anacardium*, *E. fischeriana*, *C. fistula.*, *C. fistula.*, *C. fistula.,T. terretris*, *H. antidy senterica*, *S. caudata.*, *E. ribes*, *C. tiglium*, *A. fabri*, *A. lappa*, *Brown sugar*, *V. vinifera.*, *E. japonica*
medicinal materials that record efficacy	388	8.84%	*L. brevituba.*, *P. hookeri*, *C. hendersonii.*, *G. straminea.*, *D. tanguticum*, *Moschusberezovskii*, *Snow frog*, *Zebra*, *Fish*, *Beef*, *Lamb*, *MeconopsisVig*, *C. cyminum.*, *N. glandulifera.*, *Carum carvi L.*, *A. manihot*, *Anhydrite*, *Magnetitum*, *S. officinalis*, *Aurum*, *Goethitum*, *B. grunnieus*, *B. concretio*, *C. sativus*, *A. kravanh.*, *M. fragrans*, *D. aromatica*, *S. album.*	40	1.14%	*C. mukul*, *P. capitatum*, *H. leptocarpum.*, *Fried rice*, *Terracotta*, *O. erythrotis, B. aristata.、C. longa*, *Goethitum*, *P. longum.*, *L. lacca.*, *A. vasiva*, *H. antidy senterica*, *S. anacardium*, *Aurum*, *E. ribes*, *T. chebula.*, *P. emblica.*, *T. bellerica*, *T. cordifolia.*, *R. gilgitica.*, *Z. officinale.,T. terretris*, *R. phoenicolasius.*, *I. racemosa.*
Types of foods	153	3.49%	Beef, camel meat, horse meat, donkey meat, mutton, medicinal rice, fried rice, barley porridge, fried barley, barley flour, sesame oil, white mustard oil, sheep ghee, Pian ghee, cow ghee, rice, wheat, barley, oats, peas, onions, garlic, young radish, mature radish, bramble, and so on.	392	11.18%	Dried ginger, Maurice, carrots, budazhi, yellow beef, sparrows, grouse, peacocks, rabbits, walang pulp, grapes, buy berries, round cypress berries, Xiangbuxia berries, Dalal berries, 60 m, beans, black broad beans, beans, salt, miscellaneous guaracha, padanggami, riyal and so on.
Types of drinks	65	1.48%	Yogurt, cheese, buttermilk, milk, ghee, mulled wine, cane sugar wine, barley wine, brown sugar wine, honey wine, snow water, valley water, well water, brackish water, forest water*etc.*	81	2.31%	Ghee, yogurt, milk, wine, rice wine, cow’s urine, boiled water, horse’s urine, camel’s urine, sugarcane juice, enzyme, brown sugar, boiled water, cool water, Kedar water, Ba Ri Shan water, Era Dagabao oil, white mustard oil, sesame oil, yam bean root oil*etc.*

Note: * indicates *P* < 0.05, ** indicates *P* < 0.01, and *** indicates *P* < 0.001 compared to the *Four Medical Tantras* treatments.

The *Four Medical Tantras* contains a relatively large number of medicinal materials and preparations, whose efficacy is described and for which there is a unique understanding. Tibetan medicine believes that “as long as one understands the nature and flavor of medicines and how to combine them, everything can be transformed into medicinal materials, and even those toxic substances can be transformed into effective medicines” (Dengdu, 2015a). The relatively large number of foods and drinks in the *As⋅ṭāṅga Hṛdaya Saṃhitā* is ideally in line with Ayurveda’s core philosophy of “how to avoid disease and live longer” (Ciren, 2020), which emphasizes that diet and daily habits are the best choices for preventing and treating disease.

### 3.1.2 Comparison of food types

The results of the non-parametric test showed that the types of foods were not similar overall (*P* < 0.001). Through visual comparison analysis, the types of foods in the both texts do not resemble each other much. First, regarding the total number of types of foods, there is a considerable difference between the 153 foods recorded in the *Four Medical Tantras* and the 392 foods recorded in the *As⋅ṭāṅga Hṛdaya Saṃhitā*. The second point: Regarding categorizing types of foods, the *Four Medical Tantras* divides them into five main types but does not explicitly mention the categorization of fruits and salts, whereas the *As⋅ṭāṅga Hṛdaya Saṃhitā* subdivides them into six main types but does not deal with the categorization of fats and oils. The third point: Regarding the percentage of various food items, meat (49.02%) is the highest in the *Four Medical Tantras*, while the rate of vegetables (25.77%) is the highest in the *As⋅ṭāṅga Hṛdaya Saṃhitā*. Table 2 presents the details of these two sources.

**TABLE 2:** Comparative analysis of food, drinks, and medicinal materials types.

Comparison project		*Four Medical Tantras*		*As⋅ṭā $\dot{n}$ g Hṛdaya Saṃhitā*	
		Type (species)	Percentage	Type (species)	Percentage
Types of foods	*P-value*	1.8584 × 10^−24^***			
	Types of meats	75	49.02%	95	24.23%
	Types of cooking	37	24.18%	18	4.59%
	Types of fats	16	10.46%	0	0.00%
	Types of grains	15	9.80%	55	14.03%
	Types of vegetables	10	6.54%	101	25.77%
	Types of fruits	0	0.00%	68	17.35%
	Types of salt	0	0.00%	55	14.03%
Types of drinks	*P-value*	0.029074*			
	Types of milk	34	52.31%	21	25.93%
	Types of wine	21	32.31%	19	23.46%
	Types of water	10	15.38%	14	17.28%
	Types of sugarcane	0	0.00%	16	19.75%
	Types of vegetable oils	0	0.00%	11	13.58%
Number of medicinal materials	*P-value*	7.6203 × 10^−8^***			
	medicinal materials that record efficacy	388	11.63%	40	2.52%
	Prescription medicinal materials	1,103	33.07%	752	47.48%
	medicinal materials duplicated in the two categories above	729	21.86%	30	1.89%
	Deletion of duplicates in the above two categories of medicinal materials	1,115	33.43%	762	48.11%
Types of medicinal materials	*P-value*	7.4499 × 10^−48^***			
	Botanical medicinal materials	427	38.23%	225	29.53%
	Animal medicinal materials	479	42.88%	110	14.44%
	Mineral medicinal materials	124	11.19%	32	4.20%
	Auxiliary medicinal materials	58	5.19%	47	6.17%
	The others	27	2.51%	348	45.67%

Note: * indicates *P* < 0.05, ** indicates *P* < 0.01, and *** indicates *P* < 0.001 compared to the *Four Medical Tantras* food, drinks,and medicinal materials types.

A comparative study shows various vegetables, fruits, and grains described in the *As⋅ṭāṅga Hṛdaya Saṃhitā wi*th distinctive characteristics. The types of rice are further subdivided into 32 species. This categorization is related to the vast plains and river basin topography of the Indian subcontinent, its abundant arable land, and favorable conditions for economic development (Qunpei, 2019).

### 3.1.3 Comparison of drink types

The results of the non-parametric test showed that the types of drinks in both texts were not similar overall (*P* < 0.001). Visualization comparison analysis shows a clear difference between the types of drinks. First, regarding the overall types of drinks, there are fewer drinks in the *Four Medical Tantras* (65 types) relative to the 81 types in the *As⋅ṭāṅga Hṛdaya Saṃhitā*, but the number of Dairy products is the largest in both drinks. The second point concerns categorizing the types of drinks. The drinks described in the *Four Medical Tantras* are mainly classified in to three main types: dairy products, alcoholic drinks, liquid drinks, *etc.* These categorizations are both concise and easy to understand. The book, the *As⋅ṭāṅga Hṛdaya Saṃhitā* is subdivided into five main types, with 16 more sugarcane types and 11 more sesame oil types than the *Four Medical Tantras*. Table 2 presents the details of these two sources.

By comparing the different types of drinks, we find that the number and categorization of drinks in the *As⋅ṭāṅga Hṛdaya Saṃhitā* exceeds that of the *Four Medical Tantras*. In addition, the *Four Medical Tantras* describes some drinks with unique characteristics, such as yellow cow urine, camel urine, and elephant urine. India’s climate is characterized by a tropical monsoon with high temperatures throughout the year, so people have an excellent thirst-quenching effect on various drinks, such as sugarcane (Xiaoli, 2021).

### 3.2 Comparison of information about medicinal materials

#### 3.2.1 Comparison of the number of medicinal materials

The results of the non-parametric test showed that the number of medicinal materials in both texts was not similar overall (*P* < 0.001). Through visual comparison analysis, we find that the number of medicinal materials recorded in both texts differs. The *Four Medical Tantras* recorded 1,115 medicinal materials, of which 388 recorded efficacies. These medicinal materials include arid grass, animal, soggy grass, stone, soil, treasure, plant extract, and wood medicinal materials, totaling eight types (Qingjia and Duojie, 2021). In the *As⋅ṭāṅga Hṛdaya Saṃhitā*, 762 types of medicinal materials are recorded, but only 40 that record efficacy and these medicinal materials are not further categorized. Table 2 presents the details of these two sources.

By comparing the number of medicinal materials, we find that the number of medicinal materials recorded in the *Four Medical Tantras* is much larger than that in the *As⋅ṭāṅga Hṛdaya Saṃhitā*. They have a variety of varieties, a wide range of effects, and a long history of medicinal use.

#### 3.2.2 Comparison of medicinal material types

The results of the non-parametric test showed that the types of medicinal materials in both texts was not similar overall (*P* < 0.001). Through visual comparison analysis, the types of medicinal materials recorded in both texts are not the same. There are 1,115 medicinal materials recorded in the *Four Medical Tantras*, including 427 botanical medicinal materials, 479 animal medicinal materials, 124 mineral medicinal materials, 58 auxiliary medicinal materials, and 27 other medicinal materials. In the *As⋅ṭāṅga Hṛdaya Saṃhitā*, there are 762 medicinal materials recorded, including 225 botanical medicinal materials, 110 animal medicinal materials, 32 mineral medicinal materials, 47 auxiliary medicinal materials, and 348 others (all of which are difficult to interpret Sanskrit names for medicines). Table 2 presents the details of these two sources.

The *Four Medical Tantras* is primarily centered on animal medicinal materials. In contrast, the *As⋅ṭāṅga Hṛdaya Saṃhitā* is dominated by botanical medicinal materials, which is consistent with the Indian insistence that they do not kill and that most Indians are vegetarians (Sun, 2019).

#### 3.2.3 Comparison of taste and properties of medicinal materials

In both texts, there is no record of the taste of medicinal materials. Therefore, this study compared the descriptions of the properties of medicinal materials in these two classics and found that there are apparent differences in their theories of pharmacology.

There are pronounced differences in all three aspects of the medicinal materials’ properties, taste, and effects: 1. The differences in the taste of the medicinal materials are mainly manifested in the different arrangements of the taste, which leads to significant differences in their clinical effects. 2. There are differences in the descriptions of the properties of the medicinal materials, and they do not have precisely the same medical meanings. 3. The number of efficacy and the elements contained in them are not precisely the same, and the *Four Medical Tantras* covers only the partial efficacy and the eight elements. In contrast, compared with both texts, it includes two medicinal effects and ten holistic and local elements, adding cold and heat.

Regarding clinical application, the *Four Medical Tantras* subdivided the eight elements of medicinal effects into seventeen elements. In contrast, the *As⋅ṭāṅga Hṛdaya Saṃhitā* subdivided the 10 aspects of medicinal effects into 20 elements based on the fact that one medicinal effect can restrain one morbidity factor. In terms of coping with the 20 morbidity factors, the *As⋅ṭāṅga Hṛdaya Saṃhitā* appeared to be a bit more comprehensive than the *Four Medical Tantras* (Ciren, 2020).

#### 3.2.4 Comparison of the way medicinal materials are categorized

In the *Four Medical Tantras*, medicinal materials are categorized into 17 different types, while in the *As⋅ṭāṅga Hṛdaya Saṃhitā*, medicinal materials are subdivided into 30 different types based on their efficacy in treating diseases. Table 3 presents the details of these two sources.

**TABLE 3:** Classification methods of medicinal materials in *Four Medical Tantras* and *As⋅ṭā $\dot{n}$ ga Hṛdaya Saṃhitā*.

Title of document	Number of classifications	Basis of classification of medicinal materials	Examples of medicinal materials
*Four Medical Tantras*	1	Medicinal materials for the treatment of poisonous diseases	*M. moschiferus*, *A. tanguticum*, *T. ranunculoides*, *P. trichoglossa*, *U. sessilifructus.*, *U. macrophylla.*, *M. alba*, *S. baicalensis*, *P. uralense.*, *S. scandens.*, *P. radix*, *O. reniformis*, *C. longa*, *G. urnula.*, *D. trichomanoides*, *M. daurica*, *G. scabra*, *P. hookeri*, *R. sertata*, *B. kansuensis.*
	2	Medicinal materials for the treatment of cold “Béken” diseases.	*P. granatum*, *P. nigrum*, *P. longum.*, *C. frutescens*, *A. tsaoko*, *A.m kravanh.*, *C. cassia*, *C. crista.*, *C. crista.*, *E. ribes*, *R. parvifolium*, *N. damascena*, *P. nigrum*, *C. rehderiana*, *A. rivularis*, *R. japonicus.* , *Tsabs ru thaw*, *H. violaceous*, *R. thaw.*
	3	Medicinal materials for the treatment of worm diseases	*M. moschiferus*, *F. sinkiangensis*, *Z. officinale.*, *B. monosperma*, *H. niger*, *H nigfer.*, *E. ribes*, *I. lactea*, *C. chinensis*, *A. vestita.*, *D. odora*, *A. heterophyllum*, *Z. bungeanum*, *A. japonica.*
	4	Medicinal materials of purgation	*T. chebula.*, *C. tiglium, C. fistula*, *Anacardiaceae.*, *E. fischeriana*, *E. jolkinii.*, *R. palmatum.*, *E. fischeriana*, *E. jolkinii.*, *R. palmatum.*, *S. chamaejasme.*, *R. palmatum.*
	5	Medicinal materials for the treatment of blood disease	*P. indicus, Caragana Fabr.*, *K. martin*, *L. brevity.*, *A. evasive*, *P. emblica*, *C. hendersonii.*, *P. hookeri*, *R. cordifolia., L. erythrorhizon*
	6	Medicinal materials of stopping diarrhea	*L. siceraria.*, *A. marmelos*, *S. chinensis.*, *G. acid*, *E. bignoniiflora F.Muell.*, *P. depressa.*, *P. Juss.*, *L. laccar.*, *R. cordifolia.*, *D. grandiflorum.*
	7	Medicinal materials for the treatment of pulmonary diseases	*B. concretio, G. uralensis.*, *V. vinifera*, *H. rhamnoides.*, *A. lappa*, *R. kirilowii.*, *T. squamiferum.*, *L. exscapa.*, *S. laubachia.*
	8	Medicinal materials induce vomiting	*A. chinensis, L. virgaurea.*, *O. elatus*, *A. calamus*, *L. aegyptiaca.*, *L. brachystachya.*, *S. moorcroftiana*, *C. carnosum, S. alba.*, *S. moorcroftiana*, *C. carnosum*, *S. alba.*
	9	Medicinal materials for the treatment of “Tripa” diseases	*S. bimaculata*, *H. pedunculosum*, *H. antidy senterica.*, *A. tanguticum*, *C. carnosum, G. straminea*, *B. aristata.*
	10	Medicinal materials for the treatment of “Béken” diseases	*P. sinensis.*, *I. racemosa.*, *C. sativum.*, *H. rhamnoides.*, *M. torquata*, *P. granatum*, *Z. officinale.*, *P. emblica*
	11	Medicinal materials for the treatment of pestilence diseases	*B. grannies*, *H. pedunculosum*, *A. tanguticum*, *R. kirilowii.*, *O. elatus*, *H. leptocarpum.*, *L. brevituba.*
	12	Medicinal materials are used to treat “rlung” diseases.	*T. cordifolia*, *R. phoenicolasius*, *A. agallocha*, *C. carvi.*, *A. lappa, C. muku*, *Z. officinale.*
	13	Medicinal materials for the treatment of worm diseases	*D. aromatica*, *B. grunnieus*, *B. concretio*, *C. stigma*, *M. torquata.*
	14	Medicinal materials for the treatment of urine diseases	*Sal ammoniac*, *Sallucidum*, *Gser gyi bye ma*, *Brachyura*, *A.m kravanh.*, *M. verticillata.*
	15	Medicinal materials for the treatment of “rLung and Béken” diseases	*A. officinarum*, *Z. officinale., F. sinkiangensis*, *Shen H. violaceous*, *A. sativum.*
	16	Medicinal materials for the treatment of “Huangshui” diseases	*B. carterii.*, *C. tora*, *A. manihot*, *K. martim*, *B. aristata.*
	17	Medicinal materials for the treatment of “rLung” diseases	*M. fragrans., brown sugar, bone.*
*As⋅ṭāṅga Hṛdaya Saṃhitā*	1	Medicinal materials for the treatment of “rLung” disease, “Béken” disease, worms’ disease, itching disease, and herpes diseases	*Ae la gnyis, du ru ka, A. lappa, gndh, D. tanguticum, N. chinensis., pa la bya ma ka, sbri ka tso ra tso tsa, C. cassia, I. racemosa., M. officinalis, bo la sthae ne ya, Sargassum, M. melanioides, Pinus, A. agallocha, C. stigma, shi ri ba sa ka, S. album, C. muku, lce pog dkr, bur na ma, kun du ru, B. malabaricum.*
	2	Medicinal materials for the treatment of febrile diarrhea disease,wound-healing, and “Tripa” diseases	*D. tanguticum, Brass, Goethite, Tourmaline, R. palmatum., R. cordifolia., A. vasiva, padma ge sar, shal ma li, Sa mang ga, Bu na ma, S. album, da ta kvi me tog, am pa sha, G. uralensis, na ma ka ra, tu ni shing, L. laccar. , kad tshu ra, S. multinervia, bil rkng, da ta ki, ka tang ka, a pstha.*
	3	Medicinal materials for the treatment of vomiting	*A. chinensis, G. uralensis, ku ba kha bo, E. japonica, Bim pa, Ga gon, Bi sha la, H. antidy., Radix, P. younghusbandii, C. melancholia., R. parvifolium, E. ribes Salix, C. frutescens, Kru trug tres, Ko sha pa ti ra, P. granatum, P. longum., Sallucidum, A. calamus, A.m kravanh., S. alba.*
	4	Medicinal materials for the treatment of leprosy disease, insect disease, edema disease, frequent micturition disease, and obesity diseases	*A sa na, Ti ni sha, Stg pa, Shwe ta ba ha, Ka ta ra, K. martim, Ban da, Shing sha ba, A dza sring ga, S. album, P. indicus, S. album, Ta la, A. agallocha, P. depressa., Pa la sha, Sa la, Kha ra pa tra, Dha ba, Ka ling ka, Sbro tog, Tsha ga krna, Bad tsha ka.*
	5	Medicinal materials for the treatment of “rLung” disease, “Béken” disease, skran disease, plague disease, pain, and hemorrhoids diseases	*H. antidy senterica., Bar ki, D. nobile, P. nigrum, A. tanguticum, Chu sgog, A.m kravanh., Pa ta, B. rapa., C. cyminum, Au ra lu, A za mo, A. chinensis, A. calamus, N. damascena, F. sinkiangensis, E. ribes, Pa shu gndh, Tsha ba lnga.*
	6	Medicinal materials for the treatment of heart disease, emaciation disease, “rLung” disease, “Tripa” disease, articular pain, coughs, and dyspnoea disease	*Bi da ri, Ae rndh, Ori shtsi kha li, Pu rna, C. melanochlora., C. melanochlora., BraNai ggyis, Kha sral, Vtsho byed lnga, Rtsa chung lnga, Salix, Hng sa pa tai, M. himalaica.*
	7	Medicinal materials for the treatment of nausea disease, leprosy disease, intoxication disease, plague disease, itching disease, frequent urination disease, wound-healing, and “Béken” diseases	*C. fistula, H. antidy senterica., Ka tig ta, Ba ta li, E. japonica, T. cordifolia, P. flaccidum, O. elatus, Be dng ka ta, Pa tha, Ci skayogs, She rgya ka, Karagy dzgnyis, B. striata., O. corniculata, Kru trug tres, Su sha byi ta, A. chinensis, Sa ha ra tsa, J. formosana.*
	8	Medicinal materials of stop diarrhoea	*A. chinensis, H. antidy senterica., A. lappa, C. melanochlora., R. parvifolium, G. uralensis., A. calamus, Rtsa ba bcu, Pinus, Ra sna, P. nigrum, Shu ta, Pus pa, Pa ta la, Vicia faba L, Honey, Sallucidum, R. japonicus., Shing nye ro.*
	9	Medicinal materials for the treatment of “rLung” disease, urinary frequency disease, and stone-like disease	*Be lan, Ta ra a ra, Spra tog, Bri sha sru sruvi rtsa, Ai ka ta, T. terretris, Sa ha ra dza ba na, Kashabraika Sha, Vdm bu, Ku sha rnam ggyis, Kuntha, Kha sral, Ba lu ka, Mo ra ta, Ku ru ta, Ka ram pa, M. verticillata, Be len ta la.*
	10	Medicinal materials for the treatment of rumpus, urinary, stone-like, swelling, obesity, “Béken” and seminal diseases	*Musk ka, Snu ha, T. chebula., T. bellerica, P. emblica, C. frutescens, Pa la sha, Dha ba, Shing sha pa.*
	11	Medicinal materials for the treatment of “Tripa,” blood diseases, thirst, vaginal diseases, healing wounds, fractures, and diarrhea	*Ny kro dha, A shwdtha, Au dumba ra, S. multinervia, Vdzam bu, Ardzu na, Ka pi ta, Kd pa la, Blag sha, M. indica, Salix, Bi ya la, Pa la sha, Nnti, J. formosana, Ka dam pa, Bi ra, G. uralensis., Ma du ka.*
	12	Medicinal materials for the treatment of head diseases	*A ba mrg, E. ribes, Z. officinale., P. longum., P. nigrum, B. aristata, Su ra la, Shi ri sha, Bri had, Shi kruvi sa bon, K. martim, Ma du kavi snying po, Sallucidum, T. fruticans, Pha bong dreg, C. tiglium, C. gladiata., C. carvi.*
	13	Medicinal materials for the treatment of “rLung” diseases	*Pinus, M. officinalis, A. lappa, Rtsa ba bcu, Ba la gnyis, Bi ra ta ri, Bi da ri.*
	14	medicinal materials for the treatment of “Béken” diseases, obesity, worms, colds, gastric distress, phlegm abnormalities, respiratory distress, wound-healing	*Ku the ra, Ka la ma la, E. ribes, Kha ra pa tra, Kd pa la, Bri sha ka rni sha, Kun ti, Brassica napus L, Ka pid thvi lo ma, R. subornatus, Mon snevu, Ka mu ka, Rkng mar, BaishamuShTha, Bu sri na, C. pteridophyllum, E. densa.*
	15	Medicinal materials for the treatment of “Tripa” diseases	*P. flaccidum, O. elatus, E. japonica, Ba sha kai lo ma, Kha srl prevu, S. hexandrum, A. cochinchi nensis., Shi ta ba ki, D. tanguticum, Ny kro dha, C. frutescens, Stai ra ranm gnyis, Pdmvi ze, Ban nay, M. himalaica.*
	16	Medicinal materials for the treatment of “Béken,” obesity, weak stomach, “rLung” disease, encephalopathy, rumpus disease, and sarcoma disease	*Ba ra na, She rgya ka, A. cochinchi nensis., C. frutescens, Mo ra ta, A. marmelos, Agy dzasraing, Bri ha ti ni rnam gnyis, Karagy dzgnyis, Dza ya gnyis, Shi kru ku sha, A. lappa, Ba ra.*
	17	Medicinal materials for the treatment of obesity, “Béken,” vaginosis, diarrhea, and toxicosis diseases	*S. multinervia, Sha ba, Ra ro tra, Pa la sha, Dzhin dzi ni, Pinus, Kd pa la, Ra sna, Ka tam pa, Chu shaing, Ae la, Ba lu, A shw ka, Pa ri, Be la mo tsa ka, S. multinervia.*
	18	Diarrhea-inducing medicinal materials	*E. fischeriana, Tri bid ta, T. chebula., P. emblica, T. bellerica, Bi sha la, Shang ki ni, Snu ha, S. multinervia, C. fistula, Ni lli ni, E. micractina, Kam bi ly, Milk, Cow urine.*
	19	Medicinal materials for the treatment of “rLung” disease, “Tripa,” Wasting Disease, Strengthening the Body, Promoting Milk, Tonic, and aphrodisiac disease	*J. formosana, R. hoenicolasius., B. concretio, Braitɑ tai rita tai, T. cordifolia, Vtsho byed bcu.*
	20	Medicinal materials for the treatment of breast and vaginal diseases	*P. capitatum. -Ham.ex D.Don, A. calamus, Kru trug tres, C. longa, B. aristata, S. anacardium, Pa tha, T. chebula. , T. bellerica, P. emblica, A. tanguticum, Aconitum pendulum, A. lappa, A.m kravanh., A. calamus*
	21	Medicinal materials for the treatment of “Béken” disease, poisoned disease, worms’ disease, leprosy disease, wound-healing	*Ark, Aral ka, B. malabaricum., E. fischeriana, Bi sha lye, R. subornatus, Ra sna, Pri sha la, A ba mrg, Ka da ri, P. granatum, Gi ru ka ra, Ai gu, Arga tsha.*
	22	Medicinal materials for the treatment of “rLung” disease, “Béken” disease, mucous diarrhea, obesity diseases, breast diseases	*A. calamus, P. capitatum., Pinus, A. officinarum, A. tanguticum, T. chebula., C. longa, B. aristata, G. uralensis., KraiSh NaparNi, H. antidy senterica., A. calamus, C. longa.*
	23	Medicinal materials for the treatment of “rLung” disease, thirst syndrome diseases, frequent urination	*Pa ru sha, T. chebula., T. bellerica, P. emblica, V. vinifera, Kd pa la,Ka ta kavi abrs bu, Ra dza dan, P. granatum, Ba ra da du.*
	24	Medicinal materials for health preserving	*Dzi bnti, P. sibiricum, Me da, Ma hva me da, Mungga, Ma sha pa rnai, Zngs rtsi ba, Dzi ba ka, G. uralensis.*
	25	Medicinal materials for the treatment of poisonous diseases, febrile diseases, and “Tripa” disease	*Anydza na, Pa li ni, N. chinensis., Pdma autbl, B. aristata, A. kravanh, Pierre ex Gagnep., G. uralensis., B. malabaricum.*
	26	Medicinal materials for the treatment of “Béken” diseases	*C. fistula, Ark, Mungga, A. hinensis, E. ciliata., P. capitatum, H. antidy senterica.*
	27	Medicinal materials for the treatment of “Tripa,” blood, thirst, and plague diseases	*D. nobile, M. himalaica., Ma du ka, S. album, P. indicus, G. uralensis., A. vasiva*
	28	Medicinal materials for treating frequent urination, urinary calculi, plagiocephaly, and fatty diseases.	*Ru sha ka, F. sinkiangensis, Spng ma, Sallucidum, O. erythrotis, Melanterite.*
	29	Medicinal materials for the treatment of “Tripa,” “Béken”“leprosy, plague, poisoning, nausea, gastrointestinal and eye disease	*Radix P. younghusbandii, B. striata., D. nobile S. album, T. cordifolia*
	30	Medicinal materials for the treatment of “Tripa” disease, “Béken” disease, Plague diseases, nausea, fever, thirst, stomach yang-raising	*T. cordifolia,C. sativum. ,C. frutescens, E. japonica,P. indicus*

Note: The names of medicinal materials and preparations that cannot be interpreted in the table are treated as Latin transcription of the original name.

In terms of the criteria for categorizing medicinal materials, the *Four Medical Tantras* mainly categorizes medicinal materials based on a particular disease, such as “medicinal materials for the treatment of poisonous diseases,” “medicinal materials used for treating cold Béken (Tibetan medicine believes that “Béken” dominates the body’s mucus and water metabolism, which corresponds to the elements of “earth and water,” and that its imbalance causes diseases that are characteristic of Tibetan medicine),” and “medicinal materials for the treatment of worm diseases,” *etc.* In the *As⋅ṭāṅga Hṛdaya Saṃhitā*, medicinal materials are categorized based on a variety of diseases, such as “medicinal materials used for treating ‘rLung’ disease (“rLung” in Tibetan medicine is the most active pathogenic factor in the “Three Causes Doctrine” of Tibetan medicine (rLung, Tripa, and Béken), and it is closely related to “gas” or “wind” energy; its imbalance causes diseases characteristic of Tibetan medicine), ‘Béken’s disease, worm disease, itching disease, and herpes diseases,” “medicinal materials for the treatment of febrile diarrhea disease, wound-healing, and ‘Tripa’ diseases (“Tripa” diseases in Tibetan medicine are a type of pathology related to the energy of fire in the Three Causes of Tibetan medicine (rLung, Tripa, and Béken),” and “medicinal materials for the treatment of vomiting.”

### 3.3 Comparison of preparations information

#### 3.3.1 Comparison of the number of preparations

The results of the non-parametric test showed that the number of preparations of both texts was not similar overall (*P* < 0.001). Through visual comparison analysis, the both texts are not similar regarding the number of preparations. The *Four Medical Tantras* describes in detail 2,678 different preparations, including 2,604 compound preparations and 74 single-prescription preparations. In the *As⋅ṭāṅga Hṛdaya Saṃhitā,* 2,240 different preparations are listed, including 2,093 compound preparations and 147 single-prescription preparations. Table 4 presents the details of these two sources.

**TABLE 4:** Comparative analysis of the dosage forms, usage,and delivery substance of preparations.

Comparison project		*Four Medical Tantras*		*As⋅ṭā $\dot{n}$ ga Hṛdaya Saṃhitā*	
		Quantities	Percentage	Quantities	Percentage
Number of preparations	*P-value*	1.5108 × 10^−10^***			
	Single-prescription	74	2.76%	147	6.56%
	Compound prescription	2,604	97.24%	2093	93.44%
Dosage forms of preparations	*P-value*	3.0962 × 10^−124^***			
	Unspecified category	842	31.44%	174	7.77%
	Powder medicine	630	23.53%	229	10.22%
	Medicinal broth	513	19.16%	798	35.63%
	Pastes	203	7.58%	646	28.84%
	Extracts	141	5.27%	9	0.40%
	Pills	134	5.00%	80	3.57%
	Ghee chemicals	73	2.73%	184	8.21%
	Suppository	55	2.05%	14	0.63%
	Anthracite	37	1.38%	24	1.07%
	Medicine wine agent	29	1.08%	**0**	0.00%
	Nasal drops	21	0.78%	4	0.18%
	Decoction	**0**	0.00%	78	3.48%
Usage of the preparations	*P-value*	2.3617 × 10^−7^***			
	Oral administration	1,331	72.06%	1,351	66.29%
	External use	395	21.39%	383	18.79%
	Eye drops	37	2.00%	61	2.99%
	Enemas	35	1.89%	111	5.45%
	Nasal inhalation	25	1.35%	73	3.58%
	Gargle	14	0.76%	29	1.42%
	Ear drops	7	0.38%	30	1.47%
	Drop throat	3	0.16%	**0**	0.00%
The delivery substance for the preparations	*P-value*	2.4128 × 10^−14^***			
	Water	191	65.41%	48	36.92%
	Wine	35	11.99%	12	9.23%
	medicinal material soup	33	11.30%	5	3.85%
	Urine type	16	5.48%	9	6.92%
	Bone soup	12	4.11%	**0**	0.00%
	Dairy product	5	1.71%	45	34.62%
	Meat soup	**0**	0.00%	7	5.38%
	Grape juice	**0**	0.00%	1	0.77%
	Drown sugar soup	**0**	0.00%	1	0.77%
	Honey Juice	**0**	0.00%	1	0.77%
	Soybean milk	**0**	0.00%	1	0.77%

Note: * indicates *P* < 0.05, ** indicates *P* < 0.01, and *** indicates *P* < 0.001 when compared to the *Four Medical Tantras* the dosage forms, usage,and delivery substance of preparations.

Comparatively speaking, the total number of preparations in the *Four Medical Tantras* and their compounded preparations is greater than those in the *As⋅ṭāṅga Hṛdaya Saṃhitā* in absolute numbers and as a percentage of the total. In the *Four Medical Tantras,* compounding follows three basic principles: ① Taste of medicinal materials as a guiding principle. ② To the taste of medicinal materials as a principle. ③ Performance of medicinal materials as a principle of compounding (Cuoni, 2011). Therefore, the preparations recorded in the *Four Medical Tantras* is rich in content and reflect its theoretical guidance.

#### 3.3.2 Comparison of preparation dosage forms

The results of the non-parametric test showed that the dosage forms of the preparations in both texts were not similar overall (*P* < 0.001). Through visual comparison analysis, the dosage forms of the agents recorded in the both texts are not similar. In the *Four Medical Tantras*, ten dosage forms of preparations are explicitly listed, of which powder medicine (630 times) is the most frequently used, followed by medicinal broth (513 times). In the *As⋅ṭāṅga Hṛdaya Saṃhitā*, ten dosage forms of preparations are explicitly listed, of which medicinal broth (798 times) is the most frequently used, followed by pastes (646 times). Table 4 presents the details of these two sources.

There are ten clearly labeled dosage forms of preparations in both texts, but differences exist in how these dosage forms are categorized. It is worth noting that the dosage form “decoction” is not recorded in the *Four Medical Tantras*, and the dosage form “medicinal liquor” is not recorded in the *As⋅ṭāṅga Hṛdaya Saṃhitā*. As can be seen from the descriptions above, tonics are frequent in both drinks. Methods of preparing tonics are described in the *Four Medical Tantras*, including the immersion method and the medicinal broth method, in which the medicinal broth requires the removal of two-thirds of the water. Cool drinks are recommended for hot diseases. Hot drinks are recommended are recommended for cold, and warm drinks for symptoms complicated by both heat and cold, reflecting the therapeutic concept of “disease-oriented” treatment. We do not find any record of this in the *As⋅ṭāṅga Hṛdaya Saṃhitā*.

### 3.3.3 Comparison of usage of the preparation

The results of the non-parametric test showed that usage of preparations in both texts was not similar overall (*P* < 0.001). Through visual comparison analysis, there are differences in the usage of preparations recorded in both texts. The main ways of usage of the preparations are described in the *Four Medical Tantras*, which include eight main types of oral administration (1,331 types), external use (395 types), and eye drops (37 types). The *As⋅ṭāṅga Hṛdaya Saṃhitā* describes in detail the ways of usage of the preparation, which are divided into seven main types: Oral administration (1,331 types), external use (395 types), and enemas (111 types), while there is no mention of the use of “drop throat (mid man)” in the *As⋅ṭāṅga Hṛdaya Saṃhitā*. Table 4 presents the details of these two sources.

A comparison of usage of preparations reveals that, although both texts mainly describe internally and external uses, there is a marked difference in the absolute number and proportion such preparations.

#### 3.3.4 Comparison of the delivery substance for the preparations

The results of the non-parametric test showed that the delivery substance for the preparations in the both texts preparations were not similar overall (*P* < 0.001). Through visual comparison analysis, the specifics of the delivery substance for the preparations are not similar between the two. In the *Four Medical Tantras*, six main delivery substances of preparations are described, of which water (191) and wine (35) are the most frequent. According to the *As⋅ṭāṅga Hṛdaya Saṃhitā*, there are ten delivery substances for the preparations, of which water (48) and dairy products (45, including milk juice, buttermilk, ghee, yogurt, *etc.*) are the most frequent. Table 4 presents the details of these two sources.

As can be seen from the descriptions above, the absolute quantity and percentage of water used as the delivery substance for the preparations in both texts are the highest. Tibetan medicine believes that plain water is the primary medicinal substance that guides the medicinal material into the patient’s body and enhances its efficacy (Jingfeng, 2002).

#### 3.3.5 Comparison of the numbers of medicinal material in preparations

The results of the non-parametric test showed that the numbers of medicinal material in preparations in both texts were not similar overall (*P* < 0.001). Through visual comparison analysis, the numbers of medicinal material in preparations recorded in the both texts were not the same. Specifically, there were 36 different numbers of medicinal materials in the *Four Medical Tantras*, of which those with the number of medicinal materials 1–15 accounted for 96.19% of the preparations. The minimum number of medicinal material in these preparations is 1, while the maximum reaches 116. Among them, 3-medicinal material preparations (368), 4-medicinal material preparations (352), and 5-medicinal material preparations (315) are the most common. There are 43 different medicinal materials in the *As⋅ṭāṅga Hṛdaya Saṃhitā*, with the least the numbers of medicinal material in preparations being one and the most reaching 73. The number of medicinal materials between 1 and 15 accounted for 92.58% of all preparations. The preparations with two medicinal materials (368), three medicinal materials (304), and four medicinal materials (255) were the most common. Table 5 presents the details of these two sources.

**TABLE 5:** Comparative analysis of the numbers of medicinal material in preparations.

The numbers of medicinal material in preparations	*Four Medical Tantras*		*As⋅ṭā $\dot{n}$ ga Hṛdaya Saṃhitā*	
	Frequency	Percentage	Frequency	Percentage
*P-value*	9.2239 × 10^−16^***			
1	74	2.76%	147	6.56%
2	198	7.39%	368	16.43%
3	368	13.74%	304	13.57%
4	352	13.14%	255	11.38%
5	315	11.76%	235	10.49%
6	248	9.26%	186	8.30%
7	291	10.87%	155	6.92%
8	221	8.25%	108	4.82%
9	129	4.82%	64	2.86%
10	108	4.03%	76	3.39%
11	75	2.80%	49	2.19%
12	72	2.69%	33	1.47%
13	52	1.94%	43	1.92%
14	45	1.68%	25	1.12%
15	28	1.05%	26	1.16%
16	14	0.52%	23	1.03%
17	17	0.63%	14	0.63%
18	12	0.45%	9	0.40%
19	6	0.22%	12	0.54%
20	9	0.34%	12	0.54%
21	8	0.30%	13	0.58%
22	5	0.19%	7	0.31%
23	4	0.15%	15	0.67%
24	7	0.26%	11	0.49%
25	4	0.15%	4	0.18%
26	3	0.11%	5	0.22%
27	3	0.11%	2	0.09%
28	2	0.07%	1	0.04%
29	1	0.04%	7	0.31%
30	0	0.00%	2	0.09%
31	0	0.00%	7	0.31%
32	1	0.04%	2	0.09%
33	0	0.00%	2	0.09%
34	1	0.04%	3	0.13%
35	1	0.04%	0	0.00%
36	1	0.04%	0	0.00%
37	0	0.00%	1	0.04%
38	0	0.00%	2	0.09%
39	0	0.00%	3	0.13%
41	1	0.04%	0	0.00%
42	0	0.00%	3	0.13%
43	2	0.07%	0	0.00%
46	0	0.00%	1	0.04%
48	0	0.00%	2	0.09%
52	0	0.00%	1	0.04%
57	0	0.00%	1	0.04%
73	0	0.00%	1	0.04%
116	1	0.04%	0	0.00%
Total (times)	**2,678**	**100.00%**	**2,240**	**100.00%**

Note: Comparison with the number of medicinal materials of the preparations of the *Four Medical Tantras*, * indicates *P* < 0.05, ** indicates *P* < 0.01, and *** indicates *P* < 0.001.

By comparing the numbers of medicinal material in preparations with that of the *As⋅ṭāṅga Hṛdaya Saṃhitā*, it was found that the number and the maximum numbers of medicinal material the common preparations in the *Four Medical Tantras* exceeded that of the *As⋅ṭāṅga Hṛdaya Saṃhitā*. The increase in the number of tastes of the preparations implies that they treat a more comprehensive range of diseases. Therefore, the efficacy and scope of treatment of the preparations in the *Four Medical Tantras* are more comprehensively described than those in the *As⋅ṭāṅga Hṛdaya Saṃhitā*.

#### 3.3.6 Comparison of high-frequency medicinal material of preparations

The results of the non-parametric test showed that the high-frequency medicinal materials shared in the preparations of both texts were not similar overall (*P* < 0.001). Through visual comparison analysis, the high-frequency medicinal materials common to the preparations recorded in the both texts were not similar. Among the 50 most common medicinal materials, the highest frequencies recorded in the *Four Medical Tantras* were *Terminalia chebula* Retz (558 times), *Piper longum* L. (463 times), sugar (414 times), *Zingiber officinale* Rosc. (339 times), *Amomum kravanh* Pierre ex Gagnep (278 times), honey (245 times), *Phyllanthus emblica* L. (227 times), and *Piper nigrum* L. (215 times). These medicinal materials are characteristic of Tibetan medicine and are used frequently for medicinal and food purposes. The most frequently used medicinal materials in the *As⋅ṭāṅga Hṛdaya Saṃhitā* include *P. longum* L (662 times), ghee (418 times), honey (403 times), and *T. chebula* Retz (395 times), *Z. officinale* Rosc (333 times), *P. nigrum* L (303 times) and white sugar (297 times). Of these, the Ayurveda characteristic substances of administration, such as ghee, honey, and white sugar, are mainly used in clinical practice. Table 6 presents the details of these two sources.

**TABLE 6:** Comparative analysis of high-frequency medicinal materials (top 50) for preparations.

High-frequency medicinal material	*Four Medical Tantras*		*As⋅ṭā $\dot{n}$ ga Hṛdaya Saṃhitā*	
	Frequency	Percentage	Frequency	Percentage
*P-value*	0.000023***			
*Terminalia chebula* Retz.	558	11.45%	395	4.61%
*Piper longum* L.	463	9.50%	662	7.73%
White sugar	414	8.50%	297	3.47%
*Zingiber officinale* Rosc.	339	6.96%	333	3.89%
*Amomum kravanh* Pierre ex Gagnep.	278	5.70%	83	0.97%
*Apis cerana* Fabricius	245	5.03%	403	4.71%
*Phyllanthus emblica* L.	227	4.66%	198	2.31%
*Piper nigrum* L.	215	4.41%	304	3.55%
*Santalum album* L.	210	4.31%	99	1.16%
*Aucklandia lappa* Decne.	194	3.98%	111	1.30%
*Aconitum tanguticum* (Maxim.) Stapf	184	3.78%	81	0.95%
*Sallucidum*	182	3.73%	165	1.93%
*Terminalia bellerica* Roxb	158	3.24%	280	3.27%
*G1ycyrrhiza uralensis* Fisch.	150	3.08%	232	2.71%
*Halitum violaceous*	146	3.00%	81	0.95%
*Cinnamomum cassia* Presl	144	2.96%	72	0.84%
*Euphorbia fischeriana* Steud	138	2.83%	145	1.69%
*Punica granatum* L.	132	2.71%	92	1.07%
Butter	127	2.61%	418	4.88%
Ferula sinkiangensis K.M. Shen	110	2.26%	80	0.93%
*Capsicum frutescens* L.	90	1.85%	181	2.11%
*Tinospora cordifolia* Miers.	85	1.74%	96	1.12%
*Holarrhena untidy Centrica* Wall.	84	1.72%	127	1.48%
*Dendrobiun nobile* Lindl.	0	0.00%	273	3.19%
Milk	0	0.00%	268	3.13%
Sesame Oil	0	0.00%	264	3.08%
*Aegle marmelos* (L.) Correa	0	0.00%	183	2.14%
*Berberis aristata* DC	0	0.00%	162	1.89%
*Semecarpus anacardium* Linn	0	0.00%	152	1.77%
*Cassia fistula* L.	0	0.00%	140	1.63%
*Aquilaria agallocha* Roxb.	0	0.00%	140	1.63%
*Acorus calamus* L.	0	0.00%	140	1.63%
*Rubus phoenicolasius* Maxim.	0	0.00%	139	1.62%
*Polygonum capitatum* Buch. -Ham. ex D.Don	0	0.00%	130	1.52%
*Tribulus terretris* Linn	0	0.00%	129	1.51%
*Symplocos caudata* Wall.	0	0.00%	126	1.47%
*Embelia ribes* Burm f.	0	0.00%	119	1.39%
*Croton tiglium* L.	0	0.00%	116	1.35%
*Abies fabri* (Mast.) Craib	0	0.00%	113	1.32%
Brown sugar	0	0.00%	108	1.26%
*Vitis vinifera* L.	0	0.00%	107	1.25%
*Euchresta japonica* Hook.f.ex Regel	0	0.00%	106	1.24%
*Sabina tibetica* Kom.	0	0.00%	97	1.13%
Ka sha ma	0	0.00%	95	1.11%
Tin rdu ka	0	0.00%	95	1.11%
*Meconopsis torquata* Prain	0	0.00%	95	1.11%
Cattle urine	0	0.00%	94	1.10%
Sbngs pu	0	0.00%	84	0.98%
*Commiphora mukul* (Hook. ex-Stocks) Engl.	0	0.00%	79	0.92%
*Dracocephalum tanguticum* Maxim.	0	0.00%	76	0.89%
Total (times)	4,873	100.00%	8,565	100.00%

Note: * indicates *P* < 0.05, ** indicates *P* < 0.01, and *** indicates *P* < 0.001 when compared with high-frequency medicinal materials from the *Four Medical Tantras* preparation.

Among the top 50 medicinal materials in terms of frequency, 23 medicinal materials, or 46%, were recorded in the *Four Medical Tantras*. The *As⋅ṭāṅga Hṛdaya Saṃhitā* recorded 50 medicinal materials, accounting for 100%, and the frequency of high-frequency medicinal materials was higher than that in the *Four Medical Tantras*, so it was concluded that the use of medicinal materials in the *As⋅ṭāṅga Hṛdaya Saṃhitā* was more concentrated than that of the *Four Medical Tantras*.

#### 3.3.7 Comparison of the pattern of combined use of medicinal material in preparation

**FIGURE 1:**
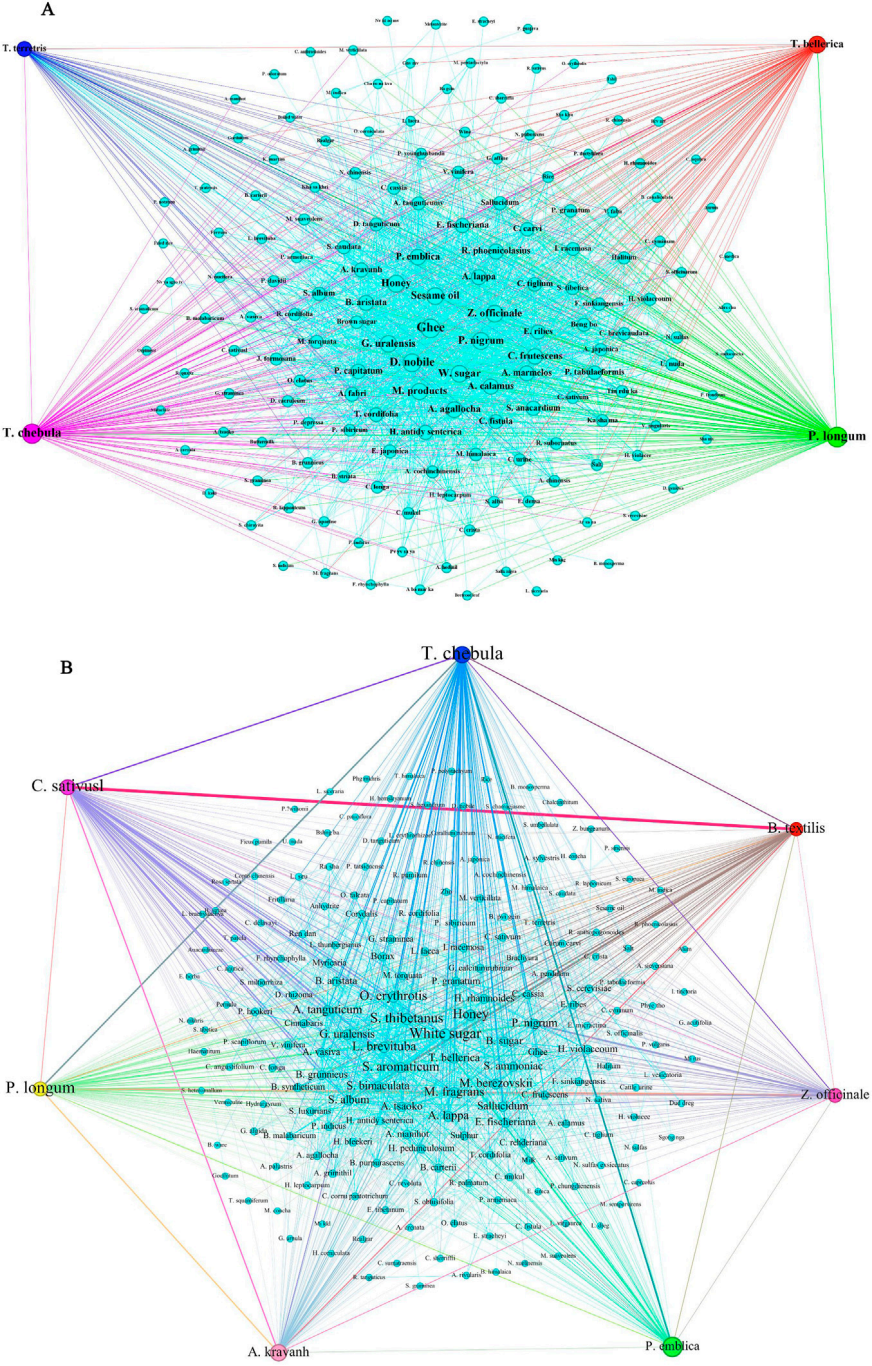
Network diagram of associations of medicinal materials in preparations recorded in the both texts (edge weight ≥6).

The results of the non-parametric test showed that the pattern of combined use of medicinal material in the preparation of both texts was not similar overall (*P* < 0.001). Through visual comparison analysis, there is not much similarity between the principles of preparation and compounding in both texts. Under the premise of “minimum conditional support of 5%, minimum rule confidence of 20%, and maximum number of antecedents of 3”, a total of 228 combinations of commonly used medicinal materials were obtained from the *Four Medical Tantras*, of which 173 were combinations of two medicinal materials, 42 were combinations of three medicinal materials, and 13 were combinations of four medicinal materials. In the *As⋅ṭāṅga Hṛdaya Saṃhitā*, we found 249 commonly used medicinal material combinations, of which 185 were composed of two medicinal material combinations, and 55 were composed of three medicinal material combinations. In comparison, nine were combinations for four medicinal material. Figure 1 and Table 7 presents the details of these two sources.

**TABLE 7:** Comparison of association rules for high-frequency medicinal material (top 20).

Association rules of medicinal material			*Four Medical Tantras*		*As⋅ṭā $\dot{n}$ ga Hṛdaya Saṃhitā*	
*P-value*			0.006856**			
Medicinal material B	Medicinal material A	Frequency	Degree of support%	Degree of confidence%	Degree of support%	Degree of confidence%
*P. nigrum*	*P. longum*	526	0	0	23.52	37.45
*Z. officinale*	*P. longum*	526	0	0	23.52	34.41
*Butter*	*P. longum*	526	0	0	23.52	25.67
*T. chebula*	*P. longum*	526	0	0	23.52	23.19
*P. emblica*	*T. chebula*	495	0	0	19.24	26.06
*P. longum*	*T. chebula*	495	0	0	19.24	24.24
*C. sativusl*	*T. chebula*	495	19.24	22.02	0	0
*B. grunnieus*	*C. sativusl*	439	17.06	21.87	0	0
*S. album*	*C. sativusl*	439	17.06	25.51	0	0
*S. thibetanus*	*C. sativusl*	439	17.06	26.65	0	0
*S. aromaticum*	*C. sativusl*	439	17.06	38.72	0	0
*A. kravanh*	*C. sativusl*	439	17.06	21.87	0	0
*White sugar*	*C. sativusl*	439	17.06	26.42	0	0
*B. concretio*	*C. sativusl*	439	17.06	56.49	0	0
*T. chebula*	*C. sativusl*	439	17.06	24.83	0	0
*Sallucidum*	*P. longum*	421	16.36	20.19	0	0
*P. nigrum*	*P. longum*	421	16.36	38.48	0	0
*Z. officinale*	*P. longum*	421	16.36	44.89	0	0
*A. kravanh*	*P. longum*	421	16.36	24.23	0	0
*T. chebula*	*P. longum*	421	16.36	28.5	0	0

Note: * indicates *P* < 0.05, ** indicates *P* < 0.01, and *** indicates *P* < 0.001 when compared with of association rules for high-frequency medicinal materials from the *Four Medical Tantras* preparation.

Among the top 20 medicinal material combinations according to support, *C*. *sativus*, *Terminalia chebula*, *B*. *grunnieus*, *S*. *album*, *S*. *thibetanus*, and *S*. *oblata* were the most strongly associated medicinal materials in the formula medicinal material combinations of the *Four Medical Tantras*. *Piper nigrum*, *Zingiber officinale*, ghee, *T*. *chebula* and *P*. *emblica* were the *As⋅ṭāṅga Hṛdaya Saṃhitā*'s most strongly associated medicinal materials.

#### 3.3.8 Comparison of the primary therapeutic diseases of the preparations

The results of the non-parametric test showed that the primary therapeutic diseases treated by the preparations of both texts were not similar overall (*P* < 0.001). Through visual comparison analysis, the two preparations differ greatly regarding the primary therapeutic diseases treated. In the *Four Medical Tantras*, the Tibetan medicine characteristic diseases were treated most frequently, 568 times, followed by digestive system diseases, 498 times, and “injuries, poisoning and certain other consequences of external causes,” 438 times. Among the preparations in the *As⋅ṭāṅga Hṛdaya Saṃhitā*, digestive system diseases (801 times) were the most frequently treated, followed by “symptoms, signs and clinical and laboratory abnormalities not classified elsewhere” (649 times) and respiratory system diseases (470 times). Table 8 presents the details of these two sources.

**TABLE 8:** Categorical analysis for comparison of the primary therapeutic diseases of the preparations.

Classification of diseases	*Four Medical Tantras*		*As⋅ṭāṅga Hṛdaya Saṃhitā*	
	Frequency	Percentage	Frequency	Percentage
*P-value*	1.8554 × 10^−59^***			
Featured Diseases	568	17.01%	372	8.20%
Diseases of the digestive system	498	14.91%	801	17.67%
Injuries, poisoning, and inevitable other consequences of external causes	438	13.11%	205	4.52%
Symptoms, signs, and clinical and laboratory abnormalities not classified elsewhere	348	10.42%	649	14.31%
Certain infectious and parasitic diseases	257	7.69%	307	6.77%
Respiratory diseases	227	6.80%	470	10.37%
Diseases of the genitourinary system	219	6.56%	363	8.01%
Diseases of the skin and subcutaneous tissue	208	6.23%	343	7.57%
Diseases of the circulatory system	138	4.13%	241	5.32%
Diseases of the musculoskeletal system and connective tissue	118	3.53%	52	1.15%
Diseases of the eye and appendages	87	2.60%	248	5.47%
Neoplasms	74	2.22%	194	4.28%
Diseases of the nervous system	60	1.80%	66	1.46%
Diseases of the ear and mastoid process	38	1.14%	40	0.88%
Pregnancy, childbirth, and puerperium	35	1.05%	70	1.54%
Mental and behavioral disorders	23	0.69%	112	2.47%
Endocrine, nutritional, and metabolic diseases	4	0.12%	1	0.02%
Total (times)	3,340	100.00%	4,534	100.00%

Note: * indicates *P* < 0.05, ** indicates *P* < 0.01, *** indicates *P* < 0.001 when compared with the main diseases treated by the *Four Medical Tantras* preparations.

As can be seen from the descriptions above, digestive system-related disorders make up a relatively high percentage of both the *Four Medical Tantras* and the *As⋅ṭāṅga Hṛdaya Saṃhitā*. Diseases of the digestive system occupy a central place in the Tibetan medical tradition, and the *Four Medical Tantras* repeatedly emphasize the preventive and therapeutic approaches to gastric disorders, with the conviction that only a physician who can diagnose and treat gastric disorders systematically is a true healer (Zhaxi, 2014). The food, dietary, and behavioral habits of the Indian population put a strain on their digestive system. Therefore, the medicinal material formulas in the *As⋅ṭāṅga Hṛdaya Saṃhitā* are practical for treating the digestive system (Sen and Chakraborty, 2017).

### 3.4 Comparative analysis of shared diets, medicinal materials, and preparations

#### 3.4.1 Overall comparison of shared treatments

**FIGURE 2:**
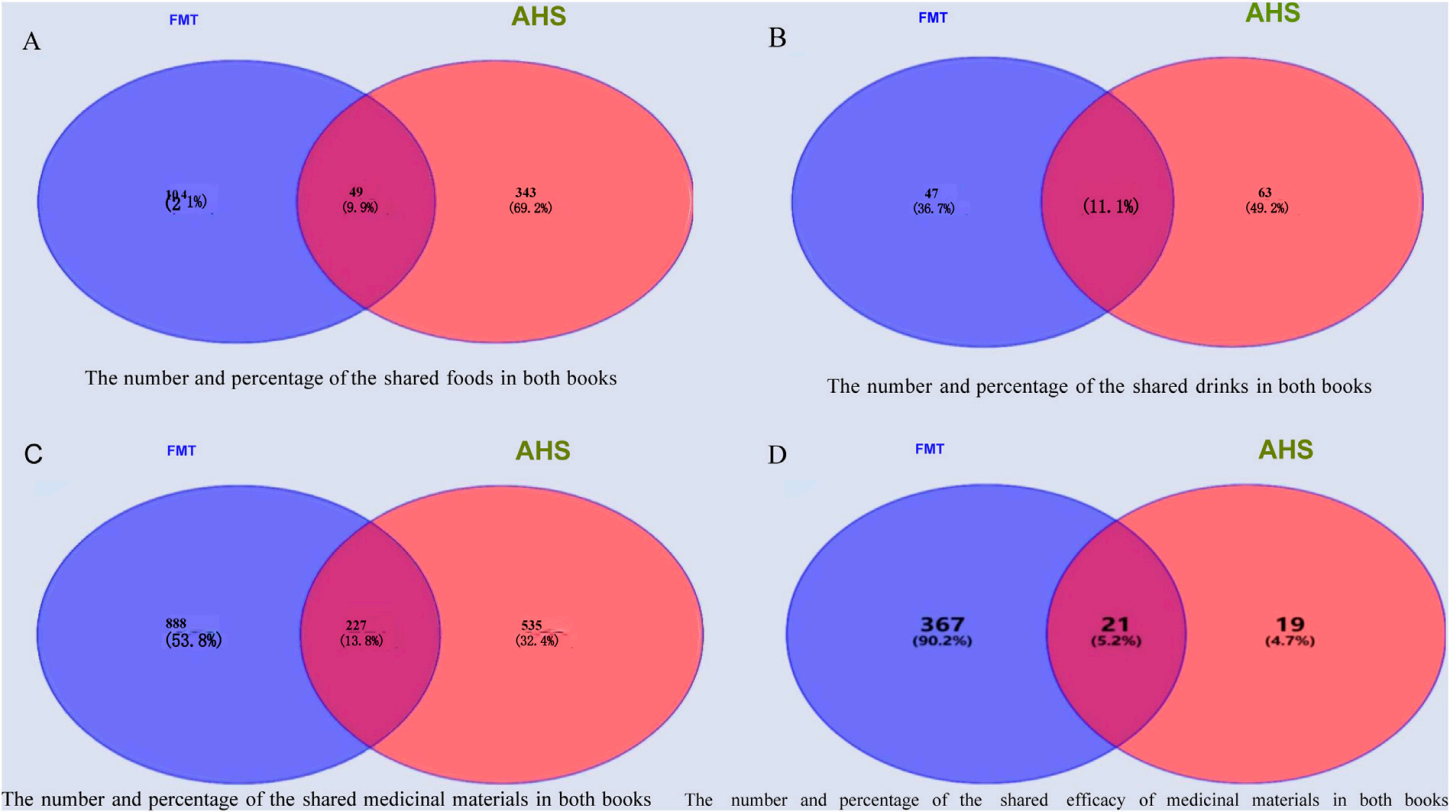
The number and percentage of the shared foods, drinks, and medicinal materials recorded in both texts together (FMT: *Four Medical Tantras*. AHS: *As⋅ṭā $\dot{n}$ ga Hṛdaya Saṃhitā*).

In both texts, 49 foods are described as treatments, which take up 9.9% of the total. A total of 18 drinks are described, accounting for 14.1% of the total. There are 227 different medicinal materials, which take up 13.8% of the total, and 21 medicinal materials whose efficacy is documented, which take up 5.2% of the total. Figure 2 presents the details of these two sources.

Through visual comparison analysis, the percentage of treatments in the both texts is not the same. For example, the rate of food types (49.02%) is the highest in the *Four Medical Tantras*, whereas the rate of medicinal material types (52.50%) is the highest in the *As⋅ṭāṅga Hṛdaya Saṃhitā.*

#### 3.4.2 Enumeration of shared diets and medicinal materials

**FIGURE 3:**
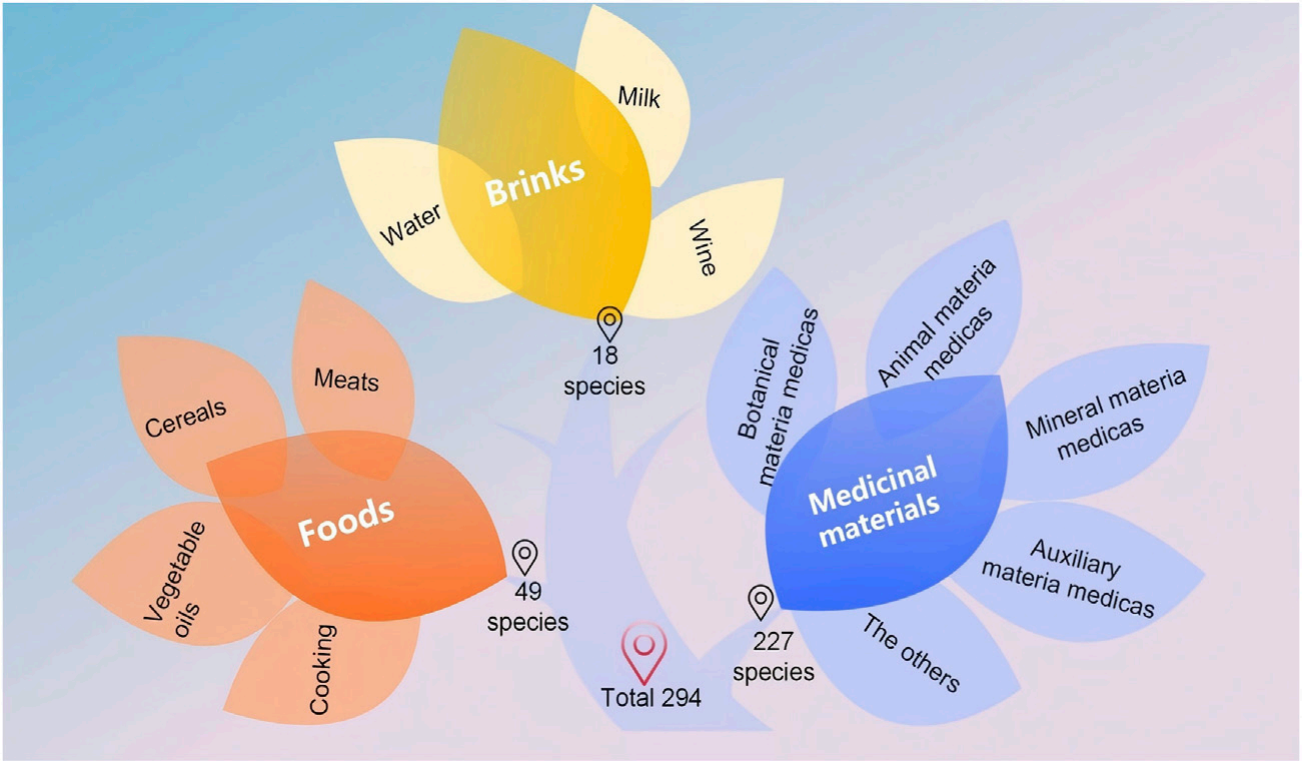
Quantity of the same foods, drinks, and medicinal materials recorded in the *Four Medical Tantras* and the *As⋅ṭā $\dot{n}$ ga Hṛdaya Saṃhitā*.

There are 49 kinds of foods recorded in both texts, which include 33 kinds of meat, eight kinds of grains, six kinds of cooking methods, and two kinds of vegetables, *etc.* A total of 18 kinds of drinks, including ten kinds of milk, six kinds of aqueous liquids, and two kinds of wines, *etc.* There are also 227 kinds of medicinal materials, including 133 kinds of botanical medicinal materials, which account for 58.85% of the total number, 51 kinds of animal medicinal materials, which account for 22.57% of the total number, and 25 kinds of mineral medicinal materials, which account for 11.06% of the total number. Figure 3 and Table 9 presents the details of these two sources.

**TABLE 9:** Types, quantities, and examples of shared diets and medicinal materials.

Categorization	Typology	Kind	Percentage	Total food enumeration
Type of food	Types of meat	33	24.09%	Peacock, Chicken, Parrot, Cuckoo, Sparrow, Deer, Tibetan Antelope, Wild Ass, Rabbit, Wild Boar, Buffalo Rhinoceros, Horse, Hornbill, Donkey, Wild Yak, Wild Dzos, Yak, Dzos, Tiger, Leopard, Brown Bear, Black Bear, Owl, Cat, Wolf, Fox, Raven, Vulture, Otter, Snake, Snow Frog, Duck
	Cereals	8	12.90%	Peas, pinto beans, fava beans, sesame seeds, caraway, rice, corn, barley
	Types of cooking	6	12.24%	Thin porridge, thick porridge, thick porridge, broth, fried rice, fried barley
	Types of vegetable oils	2	1.83%	Seedling radish, mature radish
Types of drinks	Types of milk	10	33.33%	Cow’s milk, sheep’s milk, goat’s milk, raw milk, boiled milk, fresh milk, yogurt, fresh buttermilk, casein, cheese
	Types of water	6	28.57%	Rainwater, well water, spring water, cool water, boiled water, cold boiled water
	Types of Wine	2	7.14%	New wine, old wine
Types of medicinal materials	botanical medicinal materials	133	58.85%	*P. longum, T. chebula, Z. officinale, P. nigrum, C. sativusl, T. bellerica, P. emblica, G. uralensis, B. concretio, A. kravanh, S. album, D. nobile, A. lappa, Sesame Oil, E. fischeriana, C. frutescens, A. tanguticum, M. fragrans, S. oblata, L. brevituba, P. granatum, S. chirayita, C. cassia, H. antidy senterica, A. calamus, A. marmelos, A. agallocha, F. sinkiangensis, E. ribes, B.aristata, P. capitatum, T. terretris, T. cordifolia, A. vasica, S. caudata, R. phoenicolasius, M. torquata, S. anacardium, V. vinifera, I. racemosa, C. tiglium, R. cordifolia, C. fistula, C. sativum, H. rhamnoides, C. mukul, L. lacca, B. aristata, A. tsaoko, D. aromatica, E. japonica, C. longa, S. tibetica, B. carterii, C. carvi, M. himalaica, P. tabulaeformis, A. manihot, C. rehderiana, A. cochinchi nensis, H. leptocarpum, P. sibiricum, O. elatus, P. indicus, P. armeniaca, B. striata, D. tanguticum, S. alba, B. malabaricum, U. nuda, A. japonica., C.hendersonii, C. crista, A. grimithil, D. caeruleum, Rice, F. rhynchophylla, A. pendulum, N. chinensis, A. sativum, M. paniculata, C. cyminum, J. formosana, C. carvi, R. subornatus, M. suaveolens, V. faba, G. straminea, R.chinensis, R. lapponicum, R. gilgitica, S. indicum., A. rivularis, N. glandulifera, Goethitum, P. younghusbandii, Z. bungeanum, S. graminea, R. sativus, C. aqutica, B. monosperma, S. hexandrum, C. sherriffii, S. indicum, P. dactylifera L., L. siceraria, Q. variabilis, A. hedinil, M. horridula, L. brachystachys, S. nigra, H. barley flour, T. vernicifluum., P. vulgaris, C. chinensis, A. himalaicus, S. varians, L. usitatissimum, S. pingil, R. japonicus, R. crenulata, A. offcinarum, L. supina, S. alba oil, U. laetevirens, T. ruthenica, R. sativus, P. fructus, R. spiforme, F. esculentum.*
	Animal medicinal materials	51	22.57%	Moschusberezovskii, B. grunnieus, Cattle urine, ghee, Fresh ghee, buttermilk, goat’s milk, Whelks, Mother’s milk, Stale butter, Cholesterol, Goat meat, Ox milk, Pearl, Human skull, Malachite, Goat urine, Horse manure, Mouse droppings, Cattle manure, Goat meat, Snake, Mother-of-pearl, Peacocks, Ox, Peacock’s Guts, Pigeon poop, Fish meal, Goat ghee, Horse, Fishbone, Cheese, Pork meat, Fresh buttermilk, Goat dung, Cow horn, Cow gall, Rabbit meat, Snakeskin, Goats, Human hair, Done marrow, Dog gall, Sheep’s blood, Cat bone, Donkey bone, Deer, Cat skin, Donkey Meat, Beef
	Mineral medicinal materials	25	11.06%	Sallucidum, Halitum violaceous, Sal ammoniac, Salt, Weathered Sodium Sulfate, Natrii Sulfas Exsicc, Realgar, Native copper, Red claystone, Tsab ru tshw, Goethitum, Mercury, Concha haliotidis, gold, chalcopyrite, orpiment, Ishihana, tourmaline, Silverstone, Halloysitum Rubrum, Argentum, Plumbum Rubrum, Sulphur, ceramic tile, silver white
	Auxiliary medicinal materials	16	7.08%	White sugar, honey, ghee, brown sugar, sesame oil, wine, brewer’s yeast, boiled water, distiller’s grain, fried rice, rice swill, Fine wine, sucrose wine, maize, congee
	The others	2	0.88%	Ochotona erythrotis Buchner, dud dreg

#### 3.4.3 Effectiveness of shared medicinal materials

In both texts, although 21 medicinal materials with the same name and efficacy are recorded, there is a certain degree of similarity and difference in the efficacy of these medicinal materials. In terms of sameness, such as *H*. *leptocarpum*, which is described in both texts as being used mainly in the treatment of various plague diseases. Iron filings powder is described in both texts as being used in the treatment of swelling diseases, *etc.*, about 48% of the 21 medicinal materials have similar therapeutic efficacy. Table 10 presents the details of these two sources.

**TABLE 10:** Common medicinal materials with recorded efficacy and main effects.

Latin name	Sanskrit name	Tibetan name	Efficacy in the *Four Medical Tantras*	Efficacy in the *As⋅ṭā $\dot{n}$ ga Hṛdaya Saṃhitā*	Similarity
*H. leptocarpum*	Bar ba da	པར་པ་ཏ།	Clear Oral administration heat, detoxify, plague diseases	Plague diseases	Similar
*Iron filings powder*	Lcgs phone	ལྕགས་ཕྱེ།	Edema disease, liver poisoning, eye disease	Edema disease	Similar
*A. evasive*	Vāsaka	བ་ཤ་ཀ།	Vascular disease	Vascular disease	Similar
*H. untidy*	Kuṭa ja.	དུག་མོ་ཉུང་།	Stop diarrhea, Tripa disease	Stop diarrhea	Similar
*S. anacardium*	Bhallātaka	གོ་བྱེ།	Insecticidal, fracture, stomach, and plague diseases	Hemorrhoids	Similar
*Aurum*	Gser	གསེར།	Prolonged Life, Jumbo Toxicity	Jumbo Toxicity	Similar
*E. ribes*	Vidaṅga.	བྱི་ཏང་ག	Deworming, increase stomach fire	Deworming	Similar
*T. chebula*	Harītakī	ཨ་རུ་ར།	Nourish your body, increase stomach fire, digestive aid, rLung diseases, Tripa diseases, Béken diseases	Healing wounds, eye disease, rLung diseases, Béken diseases	Similar
*Z. officinale*	Tryūṣaṇa	སྒོག་སྐྱ།	Activating blood diseases, Béken diseases, and rLung diseases	rLung diseases, increase stomach fire	Similar
*T. terretris*	Goṣura	གཟེ་མ།	Urinary obstruction , liver disease, osteoarthritis	Urinary obstruction	Similar
*O. erythrotis*	Shilajit	བྲག་ཞུན།	Clearing stomach fire, liver fire, kidney fire, heat disease	Vaginal diseases	Dissimilar
*B. aristata*	Dāru haridrā	སྐྱེར་པ།	Detoxification, stopping diarrhea, yellow water disease (Chu ser nad)	Dysuria, obesity	Dissimilar
*C. longa*	Haridrā	ཡུང་བ།	Detoxification, fracture healing, anti-inflammatory	Dysuria	Dissimilar
*P. longum*	Pippalī	པི་པི་ལིང་།	Cold sickness	Spleen Diseases	Dissimilar
*C. mukul*	Guggulu	གུ་གུལ།	Anthrax, demonic diseases, plague diseases	High-fat disease、rLung diseases, Healing Wounds	Dissimilar
*P. emblica*	Amalaki	སྐྱུ་རུ་ར།	Tripa diseases, Béken diseases, blood diseases	Prolonged life, eye disease, healing wounds, dysuria	Dissimilar
*T. bellerica*	Bibhītakī	བ་རུ་ར།	Tripa diseases, Béken diseases, yellow water disease (Chu ser nad)	Eye disease, healing wounds	Dissimilar
*T. cordifolia*	Guḍūcī.	སླེ་ཏྲེས།	Heat rLung diseases	Gout disease	Dissimilar
*K. martim*	Seng ldeng	སེང་ལྡེང་།	Blood diseases, yellow water disease (Chu ser nad)	Hansen’s disease	Dissimilar
*R. phoenicolasius*	Kndkari	ཀཎྜ་ཀ་རི།	Heat rLung diseases	Expectoration	Dissimilar
*I. racemosa*	Puṣkara mūla	པུཥྐར་མཱུ་ལ།	Heat Béken diseases	Intercostal pain	Dissimilar

Regarding dissimilarities, for example, *O*. *erythrotis* is listed in the *Four Medical Tantras* as able to clear stomach, liver, and kidney fires and is mainly used to treat feverish diseases. Whereas the *As⋅ṭāṅga Hṛdaya Saṃhitā* states that it is helpful in the treatment of all diseases and is the primary treatment for vaginal diseases, the 21 medicinal materials with differences in efficacy account for roughly 52% of the 21 medicinal materials.

#### 3.4.4 Shared preparations - “Triphala”

Among the exchanges between Chinese and foreign traditional medical cultures, none is more notable than “Triphala” (*Terminalia chebula* Retz., *Terminalia bellerica* Roxb, *Phyllanthus emblica* Linn.), whose history of dissemination has demonstrated the remarkable characteristics of “trans-ethnicity, trans-culture, and trans-era” (Jijiaguang et al., 2012; Wu, 2021). From the viewpoint of Tibetan medicine, the three elements of “rLung,” “Tripa,” and “Béken” constitute the material foundation of the human body and are the energy and foundation necessary for the maintenance of life activities—the three medicinal materials in “Triphala” act on each of these three elements. The primary function of Tibetan medicine, “Triphala,” is to clear heat and harmonize Gi and blood. It is mainly used to treat the early and late stages of plague and fever and various illnesses caused by over-exertion. When using Tibetan bloodletting therapy to treat conditions such as plateau erythrocytosis and hypertension, “Triphala” can help separate the bad blood from the positive blood in the body before bloodletting. In this study, the highly representative “Triphala” was selected as the subject of study, and the descriptions of this preparation in both texts were compared in detail. The study results revealed that although the names, dosage forms, and modes of use of the preparations regarding “Triphala” in both texts remained consistent, they differed somewhat in terms of efficacy and primary treatment. It is described in this book, The *Four Medical Tantras*, as being used primarily for treating “plague and disordered fevers, contributing to the formation of fever.” According to the *As⋅ṭāṅga Hṛdaya Saṃhitā,* its “main effect is the healing of wounds and its use in treating eye diseases.”

**FIGURE 4:**
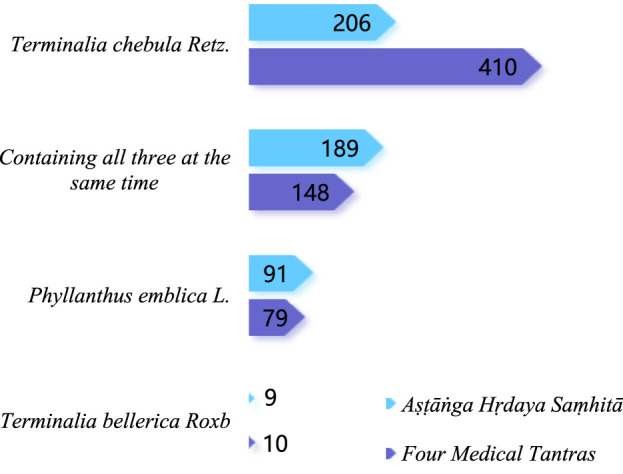
Comparative of preparations containing “Triphala”medicinal materials in the both texts.

Through visual comparison analysis, the scale of the preparations containing the three medicinal materials in both texts was not similar. In the *Four Medical Tantras*, there are 647 preparations containing the three fruits, whereas in the *As⋅ṭāṅga Hṛdaya Saṃhitā*, there are 495 preparations containing the three medicinal materials. However, the number of preparations containing all three medicinal materials in the *As⋅ṭāṅga Hṛdaya Saṃhitā* (189 species) exceeds that of the *Four Medical Tantras* (148 species). Figure 4 presents the details of these two sources.

Preparations containing only *T. bellerica* Roxb are relatively few. In the *Four Medical Tantras*, there are ten specifically, and these account for only 1.55% of the total. In the *As⋅ṭāṅga Hṛdaya Saṃhitā*, nine are recorded, accounting for only 1.82% of the total.

## 4 Discussion

For the first time, this study uses data mining techniques such as frequency statistics, association rule analysis, and complex network modeling to analyze the food, drinks, medicinal materials and preparations in the *Four Medical Tantras*, a Tibetan medical classic, and the *As⋅ṭāṅga Hṛdaya Saṃhitā*, a collection of Indian Ayurveda medical texts, in a quantitative and comparative way. It not only reveals the intrinsic characteristics of the two traditional medical systems but also provides a new academic perspective for understanding the cultural exchange between Indian and Tibetan medicine.

The Tibetan Plateau is the largest plateau in China and the highest in the world, known as the “Roof of the World” (Qiangba, 2010), and Tibetan medicine originated from the experiences of Tibetan ancestors in their struggle against nature and various diseases (Disi, 2004). Therefore, the dietary system of Tibetan medicine is clearly characterized by high-altitude environmental adaptation. The food groups recorded in the *Four Medical Tantras* were dominated by meat and fats, which accounted for 59.48% of the total food groups. The types of drinks are dominated by milk and alcohol, accounting for 84.62% of the total types of drinks. Because the Tibetan Plateau has a strong animal husbandry industry and limited crop growth, traditional Tibetan dietary therapies place special emphasis on the medicinal value of meat (e.g., yak and mutton), believing that these high-calorie foods can help the body withstand cold and replenish physical strength. Meat is often categorized as a “warm” ingredient in the Tibetan medical system and is used to treat “cold” conditions. The food items recorded in the *As⋅ṭāṅga Hṛdaya Saṃhitā* are mainly vegetables and fruits, accounting for 43.12% of the total food items. In addition to the common drinks such as milk and wine, sugar cane and sesame oil are especially recorded, accounting for 33.33% of the total drinks. Ayurvedic medicine, originating in the Indian subcontinent, is based on tropical agriculture, and its dietary therapy emphasizes the use of various vegetables, fruits, grains and legumes. Ayurveda categorizes food according to six tastes (sweet, sour, salty, bitter, spicy, and astringent) and advocates choosing the type of vegetable according to one’s constitution (dosha), for example, bitter gourd is believed to reduce fire, while pumpkin has a tonic effect. The categorization of drinks in Tibetan medicine is relatively simple, limited mainly to a few types such as ghee tea and barley wine, and focuses more on their nutritional function than on their therapeutic value. This may be related to the limited plant resources and nomadic lifestyle of the plateau region. Ayurveda, in contrast, has developed a complex system of medicinal drinks, including: 1) animal-derived drinks such as cow’s urine preparations (believed to have purifying properties); 2) fermented drinks such as coconut wine; 3) medicinal teas such as Tulsi tea; and 4) metallic infusions (e.g., gold and silver water). These special drinks are often associated with the need for high fluid and electrolyte replenishment in India’s tropical climate and reflect the importance of “purification therapy” in Indian culture.

Ethnomedicine is a multidisciplinary and comprehensive system of pharmaceutical knowledge, including natural sciences, the humanities and social sciences, and ecological and geographical disciplines (Zhang and Du, 2021). Medicine has the dual attributes of a natural science and a human science. Tibetan medicinal materials significantly reflect the characteristics of the plateau ecosystem. Animal-derived medicinal materials account for 42.88% of those recorded in the *Four Medical Tantras*, including precious substances such as musk, bear bile and antelope horn. These animal medicinal materials are believed to have powerful medicinal effects and are particularly suitable for treating common plateau diseases such as rheumatism and hypoxic diseases. Tibetan medicine also pays special attention to the use of mineral-based medicines, such as the famous prescriptions of Qishiwei zhenzhu pills and Ershiwuwei shanhu pills. Ayurveda, on the other hand, makes full use of the rich botanical diversity of the tropics. Approximately, 29.53% of the more than 762 medicinal materials recorded in the *As⋅ṭāṅga Hṛdaya Saṃhitā* are botanicals, such as wicker, turmeric, Indian ginseng, and moringa. Tropical plants have a rapid growth cycles and abundant secondary metabolites, which provide Ayurveda with a large number of biologically active botanical sources.

Tibetan medicinal preparations follow the core principle of “balance of heat and cold,” harmonizing the warm and cold properties of medicines through compounding. For example, in the preparation of valuable compound formulas such as Rinqing Changjue (Yanghong et al., 2019), the “hotness” of animal medicines is deliberately balanced with the “coolness” of plant medicines. The application of medicinal materials in the *Four Medical Tantras* preparations is characterized by a certain degree of flexibility and variability. The *As⋅ṭāṅga Hṛdaya Saṃhitā* developed a unique concept of “medicine and food,” emphasizing the synergistic effects between the two. Its preparations include decoctions of medicinal materials with food substrates such as ghee and honey (as in the famous ghee preparation) or undergo fermentation to produce asava, a liquor-like beverage. Ayurveda also emphasizes the medicinal value of everyday spices such as ginger, garlic, and cinnamon.

From the interrelation of “Diet-Efficacy-Disease” and “Drug-Efficacy-Disease” in the *Four Medical Tantras*, it can be seen that Tibetan medicine takes “cool-warm” properties of medicines as a dual core to treat the hot and cold aspects of diseases. Diseases characterized by heat and cold in both genders are seen as opposites in unity, forming an elliptical philosophical law of medication. Therefore, Tibetan medicine is characterized by “dietary therapy plus medication”, “warming the cold and cooling the hot”, and a multi-level application of concepts such as “rLung - Tripa–Béken.” The *As⋅ṭāṅga Hṛdaya Saṃhitā* is based on the “Five Elements” and the “Three Bodily Fluids (Vata, Pitta, and Kapha)” (Mohammad, 2010), emphasizing the regulating effects of the six tastes (sweet, sour, salty, pungent, bitter, and astringent) on the body, and the use of tropical plants for many of the medicinal materials. Both texts reflect the idea of ‘constitution-diet-medicine’ linkage; however, Tibetan medicine is influenced by the Buddhist principle of “cause and effect,” while Ayurveda is shaped by Hinduism’s idea of “unity of Brahman and self.” The “dietary taboos” in the *Four Medical Tantras* overlap with Buddhist precepts (Qimei, 2017), while the “detoxification therapies” in the *As⋅ṭāṅga Hṛdaya Saṃhitā* reflect the Hindu concept of purification (Tangpeng, 2006).

Due to factors such as geography and the spread of Buddhist culture, Ayurveda has influenced Tibetan medicine to a certain extent, including its fundamental theories of “Three Factors, Seven Substances, and Three Filths.” As a result, the connection between the both texts, representative classics in their respective fields of medicine, has attracted extensive attention from both domestic and international scholars. Elizabeth Fink, in her study of the *Charaka Saṃhitā*, the *Susruta Saṃhitā*, and the *As⋅ṭāṅga Hṛdaya Saṃhitā*, compared their structure and organization and pointed out that none of these three ancient Indian medical works used the same method of organization as the *Four Medical Tantras* (Wangyao, 1989). From the perspective of disease categorization, the *Four Medical Tantras* describes diseases in much more detail than the *As⋅ṭāṅga Hṛdaya Saṃhitā*. In contrast, the *As⋅ṭāṅga Hṛdaya Saṃhitā* does not mention the “Béken mubu” disease (Béken’s disease caused by blood), a key to distinguishing between heat and cold, or the “Mountain Beach Period of Fever.” In addition, the *Four Medical Tantras* categorizes fever in more detail than the *As⋅ṭāṅga Hṛdaya Saṃhitā*. Zaba and his team also noted significant differences between both texts in form and content (Zha and Dou, 2013).

In general, studies of two medical works, the both texts, have been divided into two main types: the first focuses on a comprehensive analysis of Tibetan and Indian traditional medicine and briefly explores the background of the authors of both texts, their sources, and their main contents. The second category of studies focuses on comparing the contents of both texts, exploring the deep-rooted connections between the two medical works and their historical origins. These studies are mainly qualitative and do not use quantitative research methods to make comparisons. This study is scientific and innovative in providing reference data from the dietary and pharmacologic therapeutic perspectives of both texts using a different approach than that of previous authors. Therefore, it is innovative in terms of research content and analytical tools.

Of course, this study also has its limitations, mainly reflected in the following aspects: first, the content of the study. Due to time constraints, this study only selected the parts related to dietary and medicinal materials therapy in both text. It did not cover daily living therapy and external therapy, so the therapeutic content of this study is not comprehensive. Academics have not yet conducted quantitative research on the contents of daily living therapies and external therapies. Therefore, further research in this area is needed. Second, in terms of research methodology, given the quantitative means of analysis used in this study, many non-measured factors could not be considered. We need to use qualitative analysis to validate this study’s findings. The third point concerns the data source: Since we are not well-versed in Sanskrit, we chose the Tibetan translation of the *As⋅ṭāṅga Hṛdaya Saṃhitā*, which may lead to some mismatches with the original text regarding linguistic style. Without language barriers, the best research method would be to choose the original texts of both texts: the Tibetan version of the *Four Medical Tantras* and the Sanskrit version of the *As⋅ṭāṅga Hṛdaya Saṃhitā*. The fourth point concerns data construction: given that dietary and medicinal material remedies occupy a considerable proportion of the both texts and that a large amount of the content must be entered manually, there may be missing information in the datasets, but this will not have an impact on the findings of this study.

## 5 Conclusion

A quantitative comparative analysis of the *Four Medical Tantras* and the *As⋅ṭāṅga Hṛdaya Saṃhitā* shows that there are significant differences between the two systems of medicine in the application of diets, medicinal materials and preparations. These differences reflect the distinct geographic environments, cultural backgrounds and medical theories of each system. Tibetan medicine is rooted in the ecology of the plateau, with diets and medicinal materials favoring meat and animal-based ingredients, and preparations that emphasize a balance of heat and cold. In contrast, Ayurveda relies on tropical agriculture, with a greater reliance on plant-based ingredients and a focus on synergistic medicinal food. Although their specific applications differ, both embody the wisdom of locally adapted medicine and have developed highly systematic systemic approach to treatment. It is worth noting that there is some intersection between the two systems overlap in certain foods, drinks, and medicinal materials (e.g., 9.9% of foods, 14.1% of drinks, and 13.8% of medicinal materials are the same, with a 48% similarity in the efficacy of medicinal materials), suggesting the possibility of ancient medical exchanges or a shared knowledge of certain substances with universally recognized healing properties. Future studies could further explore the pharmacological effects of these shared components and their potential application value in cross-cultural medicine. Additionally, the distinct use of drinks (e.g., animal urine) in Ayurveda and the precise regulation of cold and heat in Tibetan medicine demonstrate the unique logic of traditional medicine, which is an area worthy of reference and in-depth study in modern medicine.

## Data Availability

The datasets presented in this study can be found in online repositories. The names of the repository/repositories and accession number(s) can be found in the article/supplementary material.
